# Seagrass Radiation after Messinian Salinity Crisis Reflected by Strong Genetic Structuring and Out-of-Africa Scenario (*Ruppiaceae*)

**DOI:** 10.1371/journal.pone.0104264

**Published:** 2014-08-06

**Authors:** Ludwig Triest, Tim Sierens

**Affiliations:** Plant Biology and Nature Management, Vrije Universiteit Brussel, Brussels, Belgium; East China Normal University, China

## Abstract

Many aquatic plant and seagrass species are widespread and the origin of their continent-wide ranges might result from high gene flow levels. The response of species when extending northwards since the Last Glacial Maximum can be opposed to the structuring of their populations that survived glaciation cycles in southern regions. The peri-Mediterranean is a complex series of sea basins, coastlines, islands and river deltas with a unique history since the Messinian Crisis that potentially influenced allopatric processes of aquatic life. We tested whether vast ranges across Europe and the peri-Mediterranean of a global seagrass group (*Ruppia* species complexes) can be explained by either overall high levels of gene flow or vicariance through linking population genetics, phylogeography and shallow phylogenetics. A multigene approach identified haplogroup lineages of two species complexes, of ancient and recent hybrids with most of the diversity residing in the South. High levels of connectivity over long distances were only observed at recently colonized northern ranges and in recently-filled seas following the last glaciation. A strong substructure in the southern Mediterranean explained an isolation-by-distance model across Europe. The oldest lineages of the southern Mediterranean *Ruppia* dated back to the period between the end of the Messinian and Late Pliocene. An imprint of ancient allopatric origin was left at basin level, including basal African lineages. Thus both vicariance in the South and high levels of connectivity in the North explained vast species ranges. Our findings highlight the need for interpreting global distributions of these seagrass and euryhaline species in the context of their origin and evolutionary significant units for setting up appropriate conservation strategies.

## Introduction

Seagrasses or marine angiosperms have an important ecological role in various coastal ecosystems [1]. Seagrass meadows provide high-value ecosystem services and globally are as much threatened as mangroves or coral reefs [2] despite their vast species ranges over thousands of kilometres. Most seagrass and euryhaline genera are either tropical or temperate with *Ruppia* L. (widgeongrass) being the only ‘truly global’ representative [1]. *Ruppia maritima* L. even is putatively one of the most widely distributed flowering plants on Earth except for the highest polar regions. There is a general understanding that historical long-distance-dispersal of aquatic plants in general [3] and conservative evolutionary pathways due to the harsh marine environment [1] might explain the relatively low number of seagrass species and their large-scale distributions. However, some recently well-studied seagrasses using nuclear microsatellite markers or chloroplast haplotypes revealed a strong genetic structure for Mediterranean populations of *Posidonia oceanica* (L.) Del. [4], [5], *Cymodocea nodosa* (Ucria) Asch. [6] and *Ruppia cirrhosa* (Petagna) Grande [7], [8]. The actual distribution and genetic structure of such seagrass populations are primarily a result of historical or recent dispersal events following the Last Glacial Maximum (LGM) through propagules, either sexual through seeds or clonal by shoot fragments [8]. Additionally, their populations still might contain traces of more ancient events providing that genetic connectivity remained largely restricted to sea basins during the past millions of years.

### Large distribution ranges but restricted dispersal

Examining the historical patterns of dispersal provides insights into the response of species to changes in climate [9]. In Europe emphasis was on recent major shifts (between Atlantic and Baltic Sea), transition regions (between Atlantic and Mediterranean Sea) or vicariance (within Mediterranean Sea). Historical events that occurred during or after the LGM can be detected with fast evolving nuclear microsatellite markers [4], [5], [6], [10]. In northern Europe, all marine species now present in the Baltic Sea underwent a recent dispersal and are survivors of a more diverse postglacial flora, established during the Littorina period (8000-4000 BP) during which the Baltic Sea was more saline than today [11], [12]. Marine macroalgae and a seagrass (*Zostera marina* L.) along with many other marine organisms revealed an overall shift between Baltic and adjacent Atlantic Sea populations because of genetic diversity loss at times of dispersal events towards the Baltic [10].

The situation is however more complex in the peri-Mediterranean (Southern Europe, including northern Africa and Middle East) where temperate (e.g. *Zostera marina* [13], *Z. noltii* Hornem [14]) and tropical (e.g., *Halophila* sp. [15]) seagrasses co-occur with an endemic species, *Posidonia oceanica* [4], [5] and a near endemic which extends into neighboring Atlantic zones, *Cymodocea nodosa* [6].such that a less profound impact of the LGM can be expected than in northern Europe. During cold periods of the last Pleistocene ice age, the Siculo-Tunisian Strait barrier could cause vicariance between eastern and western Mediterranean populations due to a highly restricted gene flow as was proposed for *Posidonia oceanica* [4], [5]. The buoyant fruits of *Posidonia* should allow for LDD but even despite a theoretically expected large amount of gene flow, no extensive mixing between sea basins could be detected with microsatellites. Additionally, for a dioecious species with limited dispersal capacities, sudden shifts in clonal diversity of *Cymodocea nodosa* were observed between the Atlantic (NW Africa) and the Mediterranean populations, indicating an albeit expected low amount of gene flow [16]. Based on the evolutionary time required to accumulate many private alleles between the Atlantic and the Mediterranean, it was suggested that the colonization of NW Africa preceded the Pleistocene glaciations allowing further vicariance under glacial cycles [16]. These findings on *Posidonia* and *Cymodocea* indicate that the peri-Mediterranean seagrass populations might reflect even more ancient Pleistocene patterns than those that appeared after the LGM.

### Combining LGM, Pleistocene and MSC for shallow phylogeny

Widely distributed seagrass species thus should not be regarded *a priori* as a panmictic population as they might experience restricted gene flow in at least some parts of the world. This can be hypothesized from current hydrological or geographical barriers (major sea currents, land masses) restricting historical dispersal. Moreover, Pleistocene dispersal events along coastlines mediated by variations in sea level along with micro-evolutionary events (shallow phylogeny) might explain nowadays genetic divergences. In particular, those populations in the peri-Mediterranean that survived glaciation cycles may reveal remnant diversity hotspots [17]. Many studies tend to focus on high-latitude range expansions following the LGM. However, populations at the ‘rear edge’ of post-glacial colonization may be of higher importance for conserving genetic diversity patterns over longer time frames [18].

A drastic event in the peri-Mediterranean during the last 6 Mya was the Messinian Salinity Crisis (MSC) [19]. The transition zone between the Atlantic Ocean and the Mediterranean Sea was characterised by severe alterations when the Rifean (Gibraltar Strait) and Baetic (Southern Iberia) gateways closed [19]. The isolation from the Atlantic Ocean (5.59-5.33 Mya) led to erosion and deposition of sediments in a large ‘Lago Mare’, a series of hypersaline lakes [20] but supposed to receive freshwater in the Eastern basins from Paratethys and from major rivers, especially North African rivers, including the Nile with transient zones of brackish to saltwater. Additionally, the East Mediterranean basin separated from the West basin. Evidence for a rapid radiation and cryptic speciation following the MSC (5.2 Mya) and during the Pliocene glaciations (3.32-2.75 Mya) was found for several Mediterranean fish species [21], [22], [23], bivalves [24] and amphibians [25] using one or multiple genes from cytoplasmic DNA to identify management or evolutionary significant units, evaluate taxonomic status, phylogenetic relationships, species radiation, biogeographical events and to infer major dispersal routes following the MSC.

Most phylogenetic studies at family level demonstrate many seagrasses as old lineages dating back to the Late Cretaceous [26]. Seagrass phylogenies are mostly inferred at family or genus level by one gene e.g. *rbcL* [27], *mat*K [28] or concatenated e.g. *rbc*L with *trn*L [29], *rbc*L with *mat*K [30] and combined cpDNA-nuclear DNA approach e.g. *ITS*1, *mat*K, *rbc*L, *psb*A-*trn*H [31] but most have low resolving power for shallow phylogenies. Using multiple genes, having different evolutionary rates at introns may help to resolve the younger nodes in shallow phylogenies and be informative for phylogeography interpretation at continent-wide scales or to detect cryptic species of vicariance events. It remains important to consider the identity and the evolutionary origin of seagrasses [32] in any discussion on their diversity and distribution. In an attempt to map all seagrasses of the world, *Ruppia* could not be worked out because of deficient data on their identity and distribution [33]. The overall low level of seagrass species diversity [33], their large distribution but recently documented restricted gene flow [4], [5], [6], [7], [8] evokes questions on species identity or vicariance across different seas or distant coastlines.

*Ruppia* has a large diversity in the western part of the Mediterranean [7], [8]. Isozyme polymorphisms across European populations indicated the existence of at least three taxa *R. maritima* (2× and 4×), *Ruppia drepanensis* Tineo (2×) and *R. cirrhosa* (4×) thereby suggesting tetraploidy on basis of higher percentage of polymorphic loci [34]. Hybridization and chloroplast capture were initially detected in seven out of 38 Mediterranean populations using five cpDNA loci (*ccmp*-2, *ccmp*-3, *ccmp*-10, *trn*H-*psb*A, *rbc*L) and the nuclear *ITS*1 and *ITS*2 [7], [8]. On basis of these findings a selected subsample was used in a phylogenetic study of Meditteranean *Ruppia* [35], thereby reconfirming *R. drepanensis* as a taxon, the existence of hybrids, cp-capture and polyploids relative to *Ruppia* lineages from other continents [36] all considered within a single worldwide *R. maritima* complex including paraphyletic branches [35] using four cpDNA loci (*mat*K, *rbc*L, *rpoB*, *rpoC1*) and a nuclear locus (*phy*B). The latter view on paraphyly should be questioned because it is not yet supported by a morphological, taxonomic and nomenclatural treatment, taking into account the type specimen of *R. maritima* from the Baltic sea (Linneaus herbarium). A strong nuclear DNA divergence between *R. maritima* (ITS-A), *R. cirrhosa* (ITS-B) and *R. drepanensis* (ITS-C) may suggest the possibility of vicariance [8] or even a worldwide misconception of the *R. maritima* identity. Additionally, the cp-capture in populations of both Europe (haplogroup E in [8]) and China (haplotype A/A1 in [37] confirmed their strong divergence from *R. maritima*.

To test the hypothesis whether the actual distribution of seagrass species still might reflect a shallow phylogenetic radiation following the MSC, *Ruppia* is a good candidate because of its wide ecological amplitude in lagoon habitats when compared to the related but strictly marine habitat seagrasses. Its large distribution across major European seas and coastlines and even several inland saltwater bodies renders *Ruppia* a suitable model for investigating the following hypotheses: (1) Concordance exist between geographical distribution and shallow phylogeny; (2) The peri-Mediterranean still reflects one or more centres of origin following the Messinian Salinity Crisis; (3) The Atlantic and Baltic Sea populations (established after the Last Glacial Maximum) are a subset of the Mediterranean genetic diversity.

Therefore we report on the identity of haplogroups and their distribution for eleven cpDNA loci(as maternal lineages), two nuclear introns (for hybrid detection) and a microsatellite locus (as marker for ploidy level) in a broad geographical sample of 2843 individuals from 140 sites across Europe, the peri-Mediterranean and Africa to combine population genetics of haplotypes and shallow phylogenies. Our findings contribute to the understanding of seagrass evolution and distribution especially of the most widespread and least documented genus *Ruppia* within a biographical context of important geological changes.

## Materials and Methods

### Ethical statement

Non-destructive sampling of shoots was performed and no field studies involving the removal of rooted individuals were performed. For locations under protection, permission was obtained from local scientists and field workers who indicated sites and helped during sampling.

- Frédéric Hendoux, Director, Conservatoire botanique national de Bailleul. Locations: Platier d'Oye, Le Fort Vert, Bourbourg (Year 2008)
- Frédéric Blanchard, Director, Conservatoire botanique national sud-atlantique. Locations: Audenge, Reservoir de Pirhaillan, Marais du Conseiller, Le Teich, St Jean de Luz (Year 2008)
- Patrick Grillas, Programme director, Tour du Valat. Location: Camargue (Year 2006)
- Luka Kastelic, Secovlje Salina NP, Slovenia and Alenka Popic, Slovenian Inst. Nature Conservation, Piran: Location: Secovlje salina (Year 2008)
- Serge Müller, Chairman, Conseil scientifique Régional du Patrimoine Naturel de Lorraine, Location: Marsal (Year 2006)
- Walter Durka Helmholtz Centre for Environmental Research UFZ Halle. Location: Artern (Year 2008)
- Irmgard Blindow, Head, Biologische Station Hiddensee. Location: Hiddensee (Year 2008)

No specific permissions were required for the locations outside or neighbouring protected areas, not privately-owned or protected in any way. *Ruppia* species are not protected.

### Study sites and plant materials

*Ruppia* plants were collected in 2006–2010 in 89 coastal or inland waterbodies from Europe and the peri-Mediterranean (Table 1 and Table S1). In each site up to 30 individual shoots were collected along a 30 m transect, except for few smaller stands. A total of 2438 individual shoots were dried on silica gel and a reference herbarium for each population was deposited. Additionally, to enlarge the scope, we included peri-Mediterranean and African herbarium samples (360 individual shoots or achenes from 36 sites) from previous own field trips in 1983–1985 [34] and older voucher specimens (45 individual shoots or achenes from 15 sites - achenes were taken from different shoots or loose-lying inflorescences). All herbarium specimens are deposited at BRVU. The whole set thus consisted of 2843 samples from 140 sites regardless the species that were *a posteriori* identified as 622 samples of the *R. maritima* complex (34 sites) and 2221 *R. cirrhosa* samples (including hybrids) from 106 sites. The latter group was further analysed in detail at population level, at relevant biogeographic sea or subbasin level and at the level of two continent parts (Mediterranean versus non-Mediterranean). The considered biogeographic regions are the coastlines of the Northern Baltic Sea, Southern Baltic Sea, North Sea, Atlantic, SW Spain, Alboran, Balearic, Tyrrhenian, Adriatic, Ionian, Aegean and Levantine/Nile/Middle East subbasins. Geographical trend analysis of the within-population haplotype diversity in *R. cirrhosa* was done for 72 populations (1944 individuals collected in 2006–2010) representing a similar sampling design of transects and equal sample sizes (Table 1 and Table S1).

**Table 1:** Data collection and haplotype diversity.

	Region	Number of sites	Number of samples	Haplotypes of the *Ruppia cirrhosa* complex (including hybridization)	Gene diversity	Haplotype diversity	Mean number of pairwise differences
1:	Northern Baltic	3	50	C1	0	0	0
2:	Ostsee/Southern Baltic	6	82	C1, C2, C3, C5	0.5917	0.000195	0.6919
3:	North Sea	11	210	C1	0	0	0
4:	Atlantic	9	243	B2, C1	0.0245	0.000014	0.0489
5:	Inland Spain	4	116	A1, A2, A3, A4, A5, B1	0.5757	0.000485	1.7190
6:	Alboran/Algerian	7	181	B1, C1, C2, C4, E1	0.6449	0.001243	4.4130
7:	Balearic	20	455	B1, B3, B4, C1, C2, C3, E4	0.6326	0.000379	1.3447
8:	Tyrrhenic	15	316	B1, B2, C1, E3	0.7203	0.000798	2.8180
9:	Adriatic	5	132	B1, B2, C1	0.2164	0.000061	0.2184
10:	Ionian	11	301	B1, B2, E2	0.0133	0.000006	0.0199
11:	Northern Aegean	3	36	B1, B2	0.1571	0.000044	0.1571
12:	Levantine/Nile/Middle East	12	99	A1, B1, E1, E3, E5, E6	0.7932	0.001845	6.5530

Collection details and diversity in 12 European and Mediterranean regions of 2221 *Ruppia cirrhosa* individuals from 106 coastal lagoons and inland saline waterbodies. Additional locality details, haplotypes, ITS identity and ploidy levels of 2843 individuals of both *Ruppia cirrhosa* and *Ruppia maritima* complex are given in Table S1.

### DNA extraction, amplification and sequencing

Genomic DNA extractions were performed on dry material stored in silica gel and on herbarium materials (15–20 mg) using the E.Z.N.A. SP Plant DNA Mini Kit (Omega bio-tek). We used three cpSSR primer pairs (*Ccmp*-2, *Ccmp*-3 and *Ccmp*-10) derived from the complete sequence of tobacco (*Nicotiana tabacum* L.) chloroplast genome [38], six newly developed primer pairs *Acmp*1, *Acmp*2, *Acmp*4, *Acmp*5, *Acmp*6 and *Acmp*7 (Table S2) derived from the *Acorus calamus* L. chloroplast genome (Genbank accession number AJ879453), a non-coding region (*trn*H-*psb*A), a partially coding (*rbc*L) region [39] with primer pairs *aF-aR en cF-cR* [40] and nuclear ITS1 (primer pair *ITS*1-*ITS*2) and ITS2 introns (primer pair *ITS*3-*ITS*4) [41]. PCR amplification was carried out in 25 µL of reaction mixture containing 0.1 µL of genomic DNA (1 µL non diluted for herbarium samples), 2.5 µL 10× PCR buffer, 0.2 mM of each dNTP, 1.6 mM MgCl2, 200 nM of the forward and reverse primer, 80 µg mL-1 bovine serum albumin (BSA) and 1 U Taq DNA polymerase. The PCR reactions were performed in a thermal cycler (MJ research PTC-200 and Bio-Rad MyCycler) and started with 2 min at 94°C, followed by 30 cycles of 45 s at 92°C, 1 min at 50°C for the reactions with *ccmp*-primers, *acmp*-primers, *rbcL*-primers and *trn*H-*psb*A and 1 min at 54°C for the reactions with *ITS*-primers, 2 min at 72°C, and a final extension at 72°C for 5 minutes. Amplicon sequencing (in both forward and reverse direction) was performed by Macrogen Inc. (Seoul, South Korea).

At population level (samples of 2006–2010) a nuclear microsatellite marker (*Rupcir*3) was newly developed to estimate the 2× or 4× ploidy level after an initial check and comparison with *Phy*B (phytochrome B) marker [39], the latter showing heterologous sequences for polyploids containing two genomes of which one putatively evolved from the *R. maritima* complex [35], [36]. This microsatellite locus was isolated from *R. cirrhosa* (Spain, Estartit, Table S1) and selected from a microsatellite-enriched genomic library developed at the Vrije Universiteit Brussels following an enrichment procedure with Dynabeads [42]. Primers (F: 5′-ACCCATTTTTCTGGCCTTCT-3′; R 5′ GATACCAACCGCTTTTTCCA-3′) were designed using the same method as we applied for papyrus [43] and revealed an invariable amplification at 121 bp for all samples and a duplicated locus with alleles ranging from 172 to 205 bp for tetraploids. Polymerase chain reaction (PCR) amplification conditions were: 1.7 µl 10× PCR buffer, 2.72 µl of each 0.2 mM dNTP, 1.36 µl of 25 mM MgCl_2_, 0.68 µl 10 µM of the forward and reverse primer each (Hex-labeled), 0.17 µl of BSA (10 µg/µl), 0.17 µl of 5 U Taq DNA polymerase and 2 µl of genomic DNA, in a total volume of 17 µl. The PCR reactions were performed in a thermal cycler (MJ research PTC-200 and Bio-Rad MyCycler) and started with 3 min at 95°C, followed by 30 cycles of 45 s at 94°C, 1 min at 54°C, 2 min at 72°C and a final extension at 72°C for 5 minutes. The amplification products were denatured for 5 min at 95°C and separated on 8% denaturing polyacrylamide gels (acryl-bisacrylamide 19∶1, 7 mM urea). Fluorescence detection (Gel Scan 2000, Corbett research) and visualization with One-Dscan software (Scanalytics) were performed. Amplicon length was estimated using the Genescan 350 Tamra Size Standard (Applied Biosystems).

### Data treatment

Chloroplast DNA sequences of all *Ruppia* taxa were aligned with CLUSTAL W [44] and further adjusted manually at sites with mononucleotide repeats and insertions/deletions (indels). The up to 3555 bp long haplotypes were defined on basis of transitions, transversions, indels and mononucleotide repeats. Indels were considered as a single event and recoded as proposed by [45]. A minimum spanning network using NETWORK 4600 (Fluxus Engineering) served for haplotype definition on basis of 62 characters. A shallow phylogeny analysis (Maximum likelihood, 1000 bootstraps) of 25 haplotypes based on 49 characters (excluding the mononucleotide repeats) was performed using a GTR (General Time Reversible) substitution model with gamma correction for among-site rate heterogeneity and an estimated proportion of invariable sites. Model testing and ML tree were done in MEGA 6 [46] giving all changes equal weight.

All further population analyses of the *R. cirrhosa* complex were done on 20 haplotypes (thereby excluding populations of *R. maritima* complex haplogroup D). After omitting 37 diagnostic characters of haplogroup D, 25 characters (16 parsimonous, 6 non-parsimonous and 3 mononucleotide repeats) could be considered for these 20 haplotypes. A permutation test of the haplotype frequencies taking their mean number of differences (Dm) into account was performed for a data set either including or excluding mononucleotide repeats to verify the significance of a phylogenetic structure through comparison of G_ST_ with N_ST_ (option PERMUT) or R_ST_ (option cpSSR) with PERMUT [47]. The tests (10,000 permutations) were done at site level (N = 106). Gene diversity, nucleotide diversity, allele frequencies and non-parametric analysis of molecular variance (AMOVA) was calculated with ARLEQUIN [48]. The 106 *R. cirrhosa* sites were pooled as two groups (Mediterranean versus non-Mediterranean) and as 12 biogeographic regions. Both the conventional *F*_ST_-statistics (each haplotype treated as equally distant) and the method with pairwise differences between haplotypes were calculated. Variance was apportioned to three components (among groups, among regions within each group, within regions) for calculating fixation indices at different levels: among groups (φ_CT_); among populations among groups (φ_ST_); among populations within groups (φ_SC_) and their significance level (>1000 permutations). All analyses were performed with and without considering mononucleotide repeats.

Genetic differentiation between pairs of regions (φ_ST_), Slatkin's linearized φ_ST_/(1-φ_ST_) and gene flow estimation (N*m*) with N as the female effective population size and *m* the female migration rate were calculated with ARLEQUIN considering pairwise differences between haplotypes and 110 permutations [49]. Again, these analyses were done with and without mononucleotide regions. Slatkin's φ_ST_/(1-φ_ST_) was used for testing isolation-by-distance (IBD) between pairs of regions. The geographic distances were measured from the barycentre of a region in two ways, namely direct flight distances (across sea or land) and following nearest coastline routes. Geographic distances were log-transformed for a Mantel test using GenAlex 6.501 [50]. A generalized linear model (test for homogeneity of slopes with Tukey HSD test; critical range with Newman-Keuls test) with φ_ST_ as continuous predictor, log-km as dependent and direct flight/coastal routes as categorical, tested the difference between both routes. Negative exponential regression was used for testing the relationship between haplotype diversity of populations/regions and their latitudinal position. The threshold values for haplotype diversity and for latitudes were estimated with standard classification and regression trees (C&RT). This data mining was done at level of 72 populations (representing full transects and equal sample sizes) and at level of 12 biogeographic regions. A piecewise linear regression allowed to estimate the latitudinal breakpoint. Product-moment correlation, regression equations, GLM and C&RT were obtained with STATISTICA 9 software.

To estimate the divergence time of the *Ruppia* lineages, a phylogenetic analysis (Maximum likelihood, 1000 bootstraps) of the longest available alignment of rbcL (1091 bp) in 12 *Ruppia* (from this work and Genbank) and 24 taxa of related aquatic plant families (Genbank) was performed using a GTR (General Time Reversible) substitution model with gamma correction for among-site rate heterogeneity and an estimated proportion of invariable sites. Model testing and ML tree were done in MEGA 6 [46]. A list of taxa and Genbank numbers is provided in Table S3. Because *Ruppia* is only far related to other monocots, a large sample of related families was considered. *Acorus calamus* was used as outgroup and evolutionary rate was estimated from stem node ages of *Acorus* (134 Mya), *Cymodocea/Posidonia* (67 Mya) [26] and *Ruppia/Posidonia* (75 Mya) [51], [52], [53].

## Results

### Species identification and hybrid detection

**Figure 1:**
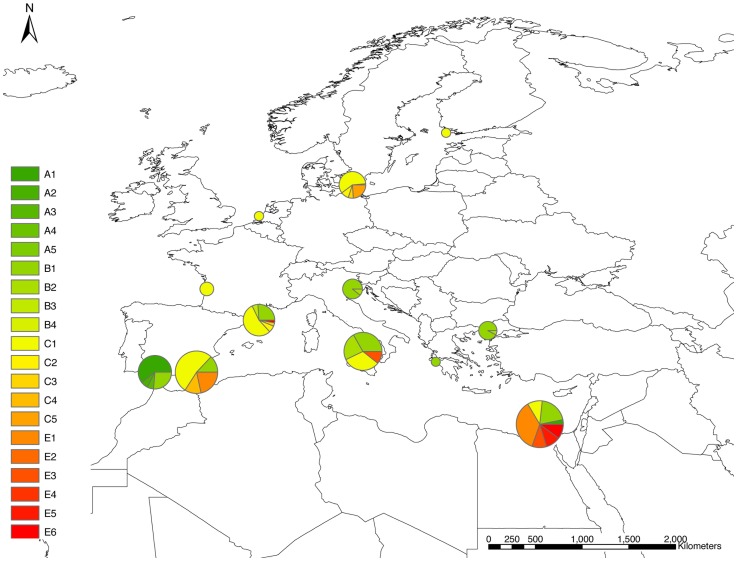
Haplotype frequency distribution. Populations of the *Ruppia cirrhosa* complex (including *Ruppia drepanensis* haplogroup A and hybrid lineages of haplogroup E) are pooled in 12 coastal zones or regions as given in Table 1 (Northern Baltic, Ostsee/Southern Baltic, North Sea, Atlantic, Inland Spain, Alboran/Algerian, Balearic, Tyrrhenic, Adriatic, Ionian, Northern Aegean, Levantine/Nile/Middle East). The size of pie charts is relative to their haplotype diversity. A detailed overview with pie charts relative to sample sizes at population level is provided in Figures S1, S2 and S3.

**Figure 2:**
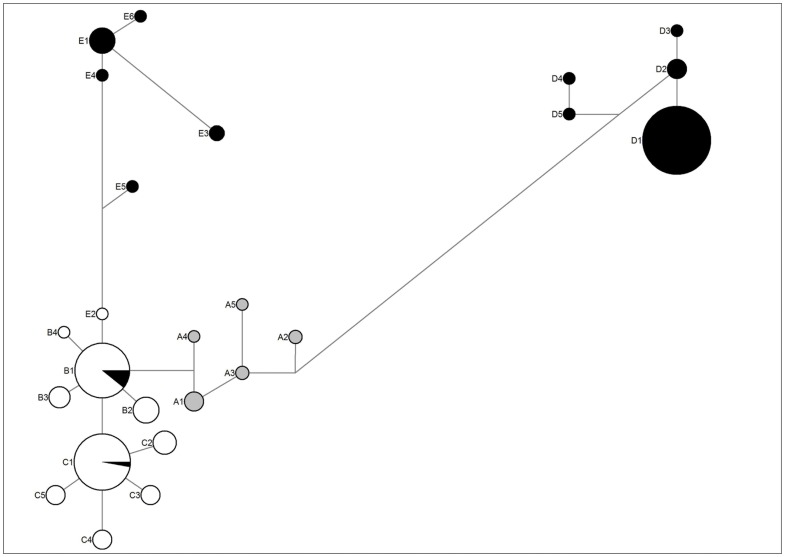
Network of 25 haplotypes. Branch lengths are relative to their differences in eleven loci (shortest branches are equivalent to a single mutation). Pie chart shading colors represent the frequency of nuclear DNA identity. Dark: ITS-A in haplogroup D (*Ruppia maritima*), haplogroup E (Ancient hybrids in *Ruppia cirrhosa* complex) and some within haplotype B1 or C1 (recent hybrids); white: ITS-B in haplogroup B and C (*Ruppia cirrhosa*); and shaded: ITS-C in haplogroup A (*Ruppia drepanensis*). Note the large number of differences between haplogroup D and E, but sharing ITS-A.

Sequencing of ten chloroplast introns and one partially coding region (rbcL) recovered 2182 and 1373 base pairs, respectively (total of 3555 bp) in two *Ruppia* species complexes (*R. maritima and R. cirrhosa*). All eleven loci revealed 25 haplotypes in 2843 individuals across Europe and Africa (Figure 1 and Figures S1, S2 and S3). The haplotypes differed in 13 transversions, 23 transitions, 13 indels and 13 mononucleotide repeats. The latter repeats were included in the network and geographic analysis but omitted from phylogenetic analysis. The most parsimonous network highlighted five groups (A, B, C, D, E) comprising three common haplotypes (B1, C1, D1). Few variants of *R. maritima* from the Baltic Sea (D2, D3) contrasted with an overall uniformity (D1) across Europe and the Mediterranean. Tropical African *R. maritima* were most divergent (D4, D5) from a common D1 (Figure 2). The divergence between haplogroup D (*R. maritima*) and all other haplogroups was largest and allowed to delineate a *R. cirrhosa* complex. A star-like network of *R. cirrhosa* haplotypes showed two kind of patterns. The widespread and common haplogroups B and C were very related whereas the rare and local haplogroups A (*R. drepanensis* Tineo) and E (undescribed taxon) showed divergent evolution. The latter (E) differed within-group for few mononucleotide repeats or even none for the former (A) in contrast to the many mononucleotide variants of B and C. The most distant haplotype within each group also differed in rbcL (i.e. A2, E3, D4, D5). Spanning the globe from the Baltic Sea to South Africa only five rbcL variants, each differing in one substitution, were found.

At nuclear DNA level both *ITS*1 and *ITS*2 regions (307 and 443 bp) were fully congruent and contained 38 informative sites in three variants ITS-A, -B and -C. The homology between these alleles reached >99% (ITS-B between haplogroups B/C and ITS-C of haplogroup A) and 95% (ITS-A of haplogroups D/E versus ITS-B or -C). *ITS*1 and *ITS*2 confirmed the nuclear divergence of the considered *R. cirrhosa* haplotype groups and additionally revealed chloroplast capture through both ancient (E) and recent (within the common B1 and C1) hybridisation (Figure 2). A nuclear microsatellite marker for polyploidy (*Rupcir*3) indicated an invariable locus for all *R. maritima* haplotypes (D), and some of the haplotypes of the *R. cirrhosa* complex (A, E) and three recent hybrid populations (within the common B1 and C1), fully concordant with the ITS information. All other haplogroups (B, C) containing a nuclear ITS-B, amplified two loci for *Rupcir*3 and confirmed their polyploidy (4×).

Overall, the *R. cirrhosa* complex from Europe and peri-Mediterranean identified on basis of 20 haplotypes thus consists of two common related haplogroups B and C, a more rare and less related haplogroup A (*R. drepanensis*), an ancient hybrid haplogroup E (consisting of several distant evolutionary lineages) and more recent introgressed hybrids with chloroplast capture. The diploid *R. maritima* complex (haplogroup D) is very distinct from the common tetraploid *R. cirrhosa* (haplogroup B, C). This information on species identity and on various hybrid origins was compulsory before conducting any phylogeographical analysis, hereafter focused on the *R. cirrhosa* complex.

### Phylogeography of the *Ruppia cirrhosa* complex

The mean number of differences between 20 haplotypes of the *R. cirrhosa* complex was Dm = 4.66 (including microsat repeats) and Dm = 4.04 (omitting repeats). A permutation test of these haplotypes in 106 sites (mean sample size of 21 individuals) revealed a significant genetic structure with Gst (0.868)<Rst (0.925) or Nst (0.923) with p<0.0001. This indicated a clear potential for a phylogenetic signal across Europe and the peri-Mediterranean.

**Figure 3:**
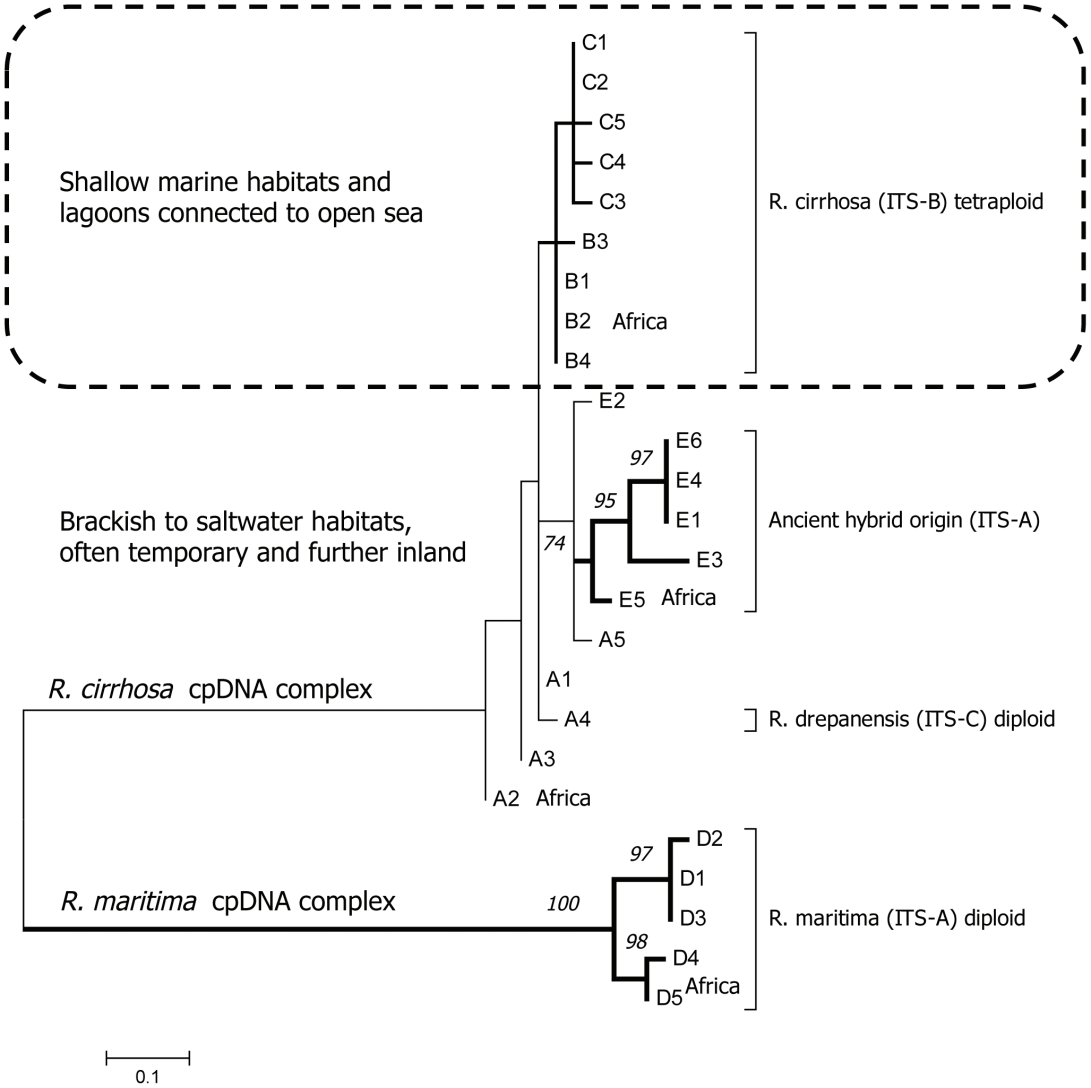
Maximum Likelihood Tree of 25 haplotypes. Two haplogroup complexes of *Ruppia cirrhosa* and of *Ruppia maritima* are fully supported. Within haplogroups, only support (>70) was given to haplotypes D and E. The basal lineages of each haplogroup contain populations of Africa. The *Ruppia cirrhosa* complex with haplogroups B and C represent tetraploid populations (ITS-B) from the most marine-lagoonal habitats whereas others haplogroups (A, D) contained diploid populations (ITS-A and ITS-C) mostly from brackish water and inland saline wetlands, including ephemeral habitats.

We tested the genetic structure at two geographical levels (Table 2). In a first approach a large scale grouping as ‘Mediterranean’ and ‘Northern Europe/non-Mediterranean’ explained nearly 20% of the variation (φ_CT_ = 0.197). About 68% of the variation resided among populations within each region. High fixation indices of the individuals within populations (φ_ST_ = 0.88, p<0.0001) and among populations within groups (φ_SC_ = 0.85, p<0.0001) indicated a strong genetic structure to unravel. In a second approach, 106 sites were *a priori* grouped to their respective coastlines (subbasins) or region for non-coastal sites (Southern Spain and SE Mediterranean). These 12 subbasins or regions showed a higher % of the variation distributed among groups (32%, p<0.0001) but still the highest proportion among the populations within groups (54.3%, p<0.0001). The fixation indices were high at all levels (p<0.0001) indicating strong structuring with a potential geographical gradient. The nucleotide diversity π reached higher values in the Southern Mediterranean than elsewhere (Rs = −0.74, p = 0.0065) with an apparent threshold at a latitude lower than 40°N situated close to the Balearic islands and Sardinia (Figure 3). A classification and regression tree analysis (C&RT) indicated a threshold at 37.5°N when considering twelve subbasins/regions and at 38.1°N when considering the population level (for this purpose 72 populations of similar sampling design). Apparently there was less impact on the haplotype diversity in the southern part of the Mediterranean as shown by the C&RT, a negative exponential curve (π = 0.029.e^−0.0635×^ with x = °N; p<0.0001) and a piecewise linear regression with breakpoint at 39°N, all values <40°N regardless the method. Both Balearic islands and Sardinia contained the highest haplotype diversity. This substructure within the Mediterranean is given by the phylogenetic more distant cpDNA haplogroups A and E. Their variants are geographically more isolated and co-occur with haplogroup B (seldom C) in the same region, however not necessarily the same site. This strong genetic structure, especially due to higher diversity in the southern Mediterranean was tested for an isolation-by-distance model using two scenarios. A first scenario considered bird-mediated dispersal using direct-flight distances between subbasins or regions (log km/*F*_ST_ Rs = 0.31, r^2^ = 0.089, p = 0.012). A second scenario followed coastal dispersal routes (either sea currents or coastal waterfowl-mediated) and revealed a clear IBD across Europe and the Mediterranean (log km/*F*_ST_ Rs = 0.50, r^2^ = 0.24, p = 0.00002). Both scenarios differed in mean distance between regions/seas (direct flight = 1692 km; coastlines = 4178 km) and their IBD strength (slope value of 0.39 and 0.48 respectively). A Mantel test showed higher significance levels for coastline distances (p = 0.004) than for direct flight distances (p = 0.02).

**Table 2:** Analysis of Molecular Variance.

Source of variation	d.f.	Sum of squares	Variance components	% of variation	Fixation indices	p-value
Among 2 groups (NW Europe and Mediterranean)	1	248.360	0.26060 σ_a_	19.66	φ_CT_: 0.197	*p*<0.0000
Among regions within each basin	104	1981.520	0.90521 σ_b_	68.30	φ_ST_: 0.880	*p*<0.0000
Within regions	2117	337.650	0.15949 σ_c_	12.03	φ_SC_: 0.850	*p*<0.0000
Among 12 Seas/regions)	11	945.291	0.39305 σ_a_	32.50	φ_CT_: 0.325	*p*<0.0000
Among Seas/regions within each group	94	1284.588	0.65687 σ_b_	54.31	φ_ST_: 0.868	*p*<0.0000
Within Seas/regions	2117	337.650	0.15949 σ_c_	13.19	φ_SC_: 0.805	*p*<0.0000

AMOVA) of *Ruppia cirrhosa* cpDNA haplotypes from 106 sites divided over two continental parts and over twelve seas or regions (d.f. = degree of freedom).

Overall, the *R. cirrhosa – complex* haplotypes thus reflect a phylogenetic signal with strong differences between northern Europe and the peri-Mediterrranean. Such higher diversity in the southern part of the Mediterranean explained an IBD model. Across Europe, coastline and sea current distances appeared more relevant than direct flight distances.

### Radiation following Messinian Salinity Crisis

**Figure 4:**
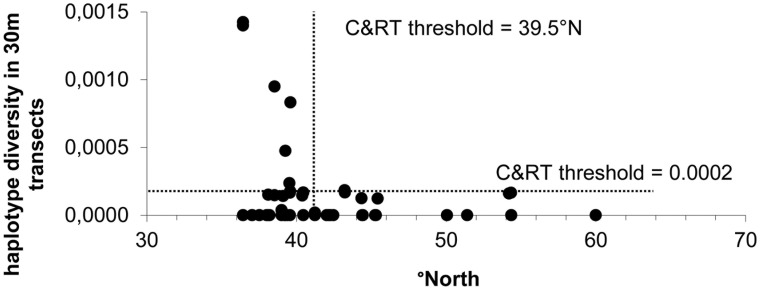
Within population diversity along latitudinal gradient. Haplotype diversity in 72 populations (30 m transects) plotted against latitudes (°N) with indication of threshold values obtained from a Classification and Regression Tree analysis. Highest within population diversity resides in regions <40°N. Note the large number of monomorph sites across all latitudes.

We further investigated whether this strong genetic structure could be explained by a recent origin of *Ruppia* haplotypes in the Mediterranean. *Firstly*, a ML of all 25 haplotypes gave insights in their topology and relationships (Figure 4). We omitted the rapidly evolving mononucleotide microsatellites from this analysis and obtained strong bootstrap support (100%) for the European (D1, D2, D3) and a more ancestral Tropical African (D4, D5) branch of the *R. maritima* complex. Within the monophyletic *R. cirrhosa* complex only the ‘ancient hybrid’ haplogroup E branch had bootstrap support above 75%. The topology indicated the Southern Mediterranean (including Northern Africa) haplotypes as ancestral to each haplogroup. Haplogroup A from Southern Spain and Northern Africa and the hybrid clade E demonstrated this as sister groups of the common haplotype B. Additionally within B, the allele variants present in Northern Africa (B1) appeared ancestral whereas the Western and Northern European haplogroup C was most derived. Shallowest nodes within the *R. cirrhosa* complex had weak phylogenetic support, particularly due to the invariable rbcL partial coding region of haplogroups B and C. *Secondly*, this phylogenetic topology also was supported by evidence from duplication events (Table S4). The presence of duplications, considered as a derived state, coincided with the overall phylogenetic outcome. *Thirdly*, a rbcL-based phylogeny of Ruppiaceae (including data from this study) and related seagrasses (from Genbank) allowed to infer the age of a *R. maritima* complex and the *R. cirrhosa* complex between 4.2 Mya and 1.5 Mya, respectively. It must be checked carefully whether the far related *R. maritima* from N. America really belongs to the same taxon as the European-African *R. maritima*, but our analysis indicate these are less related to haplogroup D than African samples to European ones (Figure S4). On basis of evolutionary rate, the European and African haplotypes undoubtedly radiated after the MSC and refilling of the Mediterranean basin. Although Ruppiaceae can be considered as an old lineage dating back to 65 Mya and far related to the seagrass families Posidoniaceae and Cymodoceaceae, we observed a recent radiation of its haplotypes corresponding to a period after the MSC. The radiation during the Pleistocene in the peri-Mediterranean thus remained reflected in the strong genetic structure and in the geographical more isolated and phylogenetic distant cpDNA haplotypes.

We finally questioned whether the subsequent glaciations and LGM influenced such pattern of older origin. In north-western Europe a high level of connectivity (Inferred gene flow levels Nm at infinity levels; p>0.05) for the *R. cirrhosa* complex was shown between the Baltic, North Sea and Atlantic, but not with most basins of the Mediterranean. In the peri-Mediterranean gene flow was very restricted (from 0.006 to 5.1 but mostly below 1 and all pairwise differentiations at p<0.05.) except for the neighbouring Adriatic Sea, and Greece (Ionian/Aegean Seas), albeit a shallow sea connection of recent origin.

## Discussion

This study gave insight in the actual continent-wide distribution of the *Ruppia* species-complexes across Europe and parts of Africa. Their genetic structure still reflects an imprint of shallow phylogenetic radiation following the MSC, with all basal clades occurring in Africa, hence supporting an ‘out-of-Africa’ hypothesis. The hotspots of diversity within some regions of the peri-Mediterranean were characterized by restricted gene flow, whereas following the LGM an expansion of a very limited number of haplotypes allowed to infer high levels of connectivity, especially along coastlines located north of the Mediterranean. We used a multigene approach in *Ruppia* and identified haplogroup lineages, ancient and recent hybrids with most of the diversity residing in the peri-Mediterranean. Data on cpDNA markers and nuclear DNA showed substantial genetic diversity within the genus *Ruppia* and revealed some well-supported clades, including highly divergent haplotype groups corresponding to two complexes (*R. maritima* and *R. cirrhosa*) across Europe and the peri-Mediterranean. The taxon *R. drepanensis* (haplogroup A) was most closely related to *R. cirrhosa*, hence considered within this complex. However, the haplotype network identified numerous gaps corresponding to a highly dynamic evolutionary pattern with many extinct steps of haplotype divergence. This points at recurrent local extinctions and limited dispersal of survivors on longer term. These taxa are also known from far inland and temporary habitats.

It was necessary to delineate those species complexes and evolutionary lineages before addressing general questions on shallow evolutionary processes and continent-wide diversity of these species closely related to seagrasses. This study then focused on the most polymorphic *R. cirrhosa* complex and supported the hypothesis that a strong substructure of *R. cirrhosa* already was present in the Mediterranean before the LGM. Centres of origin and diversification of contemporary European *Ruppia* lineages followed the MSC.

### MSC, Pleistocene and Mediterranean subbasin structure

Although the seagrass and coastal aquatic plant fossil record is very poor it allowed to indicate that angiosperms colonized marine environments about 100 Mya [54]. Fossil evidence of *Cymodocea* and *Posidonia* in Europe was known from the early Eocene (56 - 41 Mya) [55] and most seagrass lineages are represented in the late Eocene (41 - 34 Mya) [56] whereas an extinct genus *Limnocarpus* with similar fruits as *Ruppia* already was widespread in the Late Paleocene (56.0–58.7 Mya) in Europe and Asia [57], [58]. Our results using evolutionary rates of *rbcL* [23], [48], [49], [50] are in agreement with this albeit limited fossil evidence. Additionally, it follows the hypothesis of recent *Ruppia* radiation with regard to extinct ancestral genera *Limnocarpus* (until late Oligocene ca. 23 Mya) and *Midravalva* (during Miocene) that evolved from freshwater to brackishwater habitats [58]. The oldest lineages of the *R. maritima and R. cirrhosa* complex dated back to the period between the end of the Messinian and the Late Pliocene when estimated from *rbc*L seagrass phylogeny. Rapid radiation was reflected by the distribution of surviving distant haplotypes. Despite subsequent glaciations with southern range changes, an imprint of ancient allopatric origin with basal African lineages was left at peri-Mediterranean level. Phylogeographic patterns at ‘rear edges in hotspots’ might be strongly influenced by speciation processes predating the Pleistocene glaciation cycles and still reflect the origin of extant European species. The separation of cpDNA haplogroups is then expected to have persisted during the Pleistocene. Lineages older than periods before quaternary glaciations (>2.588.000 years ago) thus could have co-existed. Suitable conditions for seagrass radiation and *Ruppia* in particular (because of the adaptation of *Ruppia* members to sheltered shallow coastal lagoons and inland saline habitats) existed during and after the MSC in for example the Balearic and Levantine basins, forming large lacustrine systems that were probably interconnected [59]. A rapid radiation during the MSC brackish water or freshwater phase (around 5.5-5.3 Mya) of aquatic organisms potentially initiated in the ‘Lago Mare’. Areas with brackish water lagoons at the mouth of principal rivers (Ebro, Rhône, Pô and Nile) when dropped 1 km [60] or even 3 km [61] below the present level, could be hypothesized as isolated refugia during the MSC and thus also as potential centres of origin. A further dispersal and divergence of peri-Mediterranean *Ruppia* potentially started after the MSC (5.2 Mya) when the Strait of Gibraltar re-opened and the Mediterranean basin was refilled in a rather short period of time, ca. 100 years [16]. The transition zone between the Atlantic Ocean and the Mediterranean Sea was characterised by severe alterations when the Rifean (Gibraltar Strait) and Baetic (Southern Iberia) gateways closed: Thus, inland saline water bodies were available for radiation of haplogroups A (*R. drepanensis*) and E, nowadays adapted to shallow waters of fluctuating salinities [8]. Rapid radiation was reflected by the distribution of surviving distant haplotypes in a period after the MSC characterized by refilling of different subbasins, more precisely Alboran/Algerian, Balearic, Tyrrhenian in the West and Adriatic/Ionian, Aegean and Levantine in the East, additionally separating West and East by the Siculo-Tunisian strait as observed for other Mediterranean seagrass species [4], [5]. Haplotypes could survive within these subbasins despite subsequent glaciations with an assumed limited effect on southern range changes. Nowadays Mediterranean *Ruppia* shows a strong East-West cpDNA differentiation with most of the diversity residing in the western basin [7]. Seagrass vicariance between eastern and western Mediterranean populations also was proposed for *Posidonia oceanica* [4], [5] and between the Atlantic (NW Africa) and the Mediterranean populations of *Cymodocea nodosa* [6] using nuclear microsatellites.

Global seagrass diversity is low when considering morphological traits and phenotypes, suggesting that its species can have vast ranges along coastlines [1]. Most seagrass species indeed show relatively low chloroplast DNA variation among their populations, even over large distances, especially for *rbc*L sequence fragments mainly capable of resolving up to seagrass family and genus level whereas other cp DNA regions (e.g. *mat*K) can be more reliable in resolving species or even population level [62]. This might suggest a rather strong selection for adaptation in marine environments. Genes involved in translation, metabolism and especially photosynthesis were identified as candidate genes under positive selection for marine adaptation of *Zostera* and *Posidonia* [51], thereby potentially limiting polymorphism in coding genes such as rbcL and methodologically giving lower phylogenetic support. However, the contemporary strong substructure in the Mediterranean reflects centres of origin and at least for *Ruppia* a further diversification of lineages following the MSC. Given such recent radiation of *Ruppia*, the vicariance of several seagrass lineages, and an assumed limited impact of LGM on southern Mediterranean populations, it is less likely that high levels of recent LDD only might explain their vast species range. An alternative explanation, namely a complete (or more pronounced) mixing of genotypes (during LGM) between West and East Mediterranean basins and a regained substructure in interglacial periods, also seems highly unlikely. A possible and trivial explanation is the post-Messinian origin of *Ruppia* haplotypes reflecting on-going radiation since the Pleistocene, including various hybridization events.

### The peri-Mediterranean as a playing ground for hybridization

Hybridization events in *Ruppia* can be proposed at three time scales when interpreting our network and phylogenetic trees. A first ancient (post-Messinian) hybridization event occurred through hybridisation of male *R. maritima* (because of similar nuclear ITS) and female *R. cirrhosa* (more precisely a diploid ancestor within this complex) through chloroplast capture after repeated hybridization and further evolution towards the contemporary divergent and diverse haplogroup E. These fertile populations [8] are rare, often unique, and scattered in shallow temporary coastal lakes of the peri-Mediterranean and did not introgress further into a hybrid swarm. This taxon of ancient hybrid origin is yet unnamed. We need to obtain more detailed information on morphology and diagnostic features among the very distinct haplotypes of group E.

Second, an allotetraploid origin of *R. cirrhosa* is indicated straightforward by a duplicated nuclear microsatellite locus. Their individuals also contain two nuclear *Phy*B (Phytochrome B) sequences of which one corresponds to *R. maritima* [36]. We could confirm such heterologous *Phy*B sequences in *R. cirrhosa* and on basis of our phylogenetic tree assume the origin of such tetraploids (captured by haplotype groups B and further differentiated to C) in the Late Pleistocene. However, such heterogeneous sequences could not be detected with nuclear *ITS*1 and *ITS*2 introns, most likely due to their concerted evolution of tandem repeats [63]. Seagrasses are not uniform in the features of their functional ITS sequences and pseudogenes. Low intra-individual ITS polymorphism of pseudogenic sequences was found in the Mediterranean *Posidonia*, whereas limited homogenization of such ITS paralogues was observed in *Halophila stipulacea* (Forssk.) Asch. [64].

Third, we observed more recent introgressed hybrids between female *R. cirrhosa* (i.e. haplotype B1 or C1) and male *R. maritima* (i.e. nuclear ITS-A) or male of *R. cirrhosa* complex (i.e. haplotype E with nuclear ITS-A) at several locations (Southern Atlantic coast of France, Western Mediterranean coast of Spain and island of Menorca). Chloroplast capture was evident, although heterogeneous ITS introns again were not observed (as should be expected in F1 hybrids), assuming further local introgression or repeated backcrosses with male *R. maritima*. As a unidirectional hybridization process this appears consistent with a high amount of pollen produced by *R. maritima* and potential pollen flow towards other taxa.

Nuclear DNA alleles are more likely to confer phenotypic effects that represent adaptive responses to different ecological niches than cpDNA haplotypes. *Ruppia* is not a typical seagrass genus occurring solely in open marine environments but contains euryhaline taxa occupying several niches [1]. These thrive well in a wide variety of habitats and occurs in brackish to hypersaline pools, coastal lakes, lagoons connected to the sea or shallow open marine sites. *R. cirrhosa* may co-occur with *Zostera marina* (eelgrass) in shallow sheltered coastal zones of temperate regions [1], [8]. Because of such variety in niches at the coastal zone, even hundreds of kilometres far inland for some taxa such as *R. maritima* (sic! What's in a name), the dispersal of *Ruppia* is expected to be bird-mediated over long distances as suggested for many widespread aquatic plants [3]. Although this is a valid hypothesis for populations of the *R. maritima* complex, *R. drepanensis* (A) and the ancient hybrid (E), it was discussed and questioned for the haplotype groups B and C of the tetraploid *R. cirrhosa* sensu strictu [8]. Because the five European haplogroups of *Ruppia* differ strongly in their seed set [8] and thus consequently differ in their potential dispersal rate, a high seed rain over long distances would accelerate the decay of a phylogeographical pattern whereas both lowered local seed rain and prominent vegetative reproduction mode would indeed better safeguard ancient patterns.

Tip haplotypes of the network, also unique to a population are commonly found for waterplants and can be supposed to occur after LDD [65] or after clonal expansion. On basis of the network and the NJT we consider that highly dynamic populations of haplogroup A and E (hybrids) represent relics of diploid lineages (ITS-A). It can be hypothesized that these suffered more from bottle-necks and stochastic events (i.e. drying out of coastal pools on short term, LDD) than tetraploid populations of haplogroup B and C with nuclear ITS-B that appear to have more stable growth rates through clonal expansion usuallyin permanent lagoon systems or regularly flooded saltmarshes. Haplogroups A and E cope with higher levels of drift and stochasticity (including dispersal towards inland and temporary brackish and saltwater pools). Haplogroup E (by definition of an hybrid origin) presumably originated during Pleistocene Ages in areas where two clades formed hybrid zones (clades D and A). The highest diversity of haplogroup E and its basal lineages were found in the Nile Delta region and therefore might be hypothesized as an area of origin for these ancient hybrids. The latter haplogroup E might be a more widespread ancient hybrid in the Old World and should be monitored in further detail as it was recorded from Southern Europe [8], [35], (this study), Africa (this study) and Asia [36], [37].

### Northward expansion of a young clade (haplogroup C) after LGM

A strong substructure of high cpDNA diversity in the southern part of the peri-Mediterranean explained an isolation-by-distance model across Europe when considering Mediterranean versus non-Mediterranean populations. Strong geographic partitioning of genetic variation in the South was observed across small spatial scales (at close vicinity within coastal area or in populations at short distances) whereas *R. cirrhosa* populations from higher latitudes (>40°N) showed genetic homogeneity across vast ranges. This pattern resulting from recent colonization of Atlantic, North Sea and Baltic coastlines thus is in strong contrast to the marked phylogeographical structure of the Mediterranean subbasins. Consequently high levels of historical seed flow, reflecting northwards expansion of *R. cirrhosa* following the LGM, were only observed at mid- and northern ranges (only for haplogroup C) but equally in recently-filled seas within the Mediterranean such as the northern Adriatic Sea populations (only haplogroup B). The latter young sea is expected to show high levels of historical seed flow with populations of the Southern Adriatic and Ionian Seas or harbouring unique post-glacial marine populations or even clones as observed for *Posidonia* [66].

Atlantic/Northsea/Baltic genetic homogeneity certainly seems consistent with postglacial origins. For *Ruppia*, in contrast to *Zostera* that is only sea current dispersed [67], a number of dispersal vectors could potentially explain the northward colonization. Diploid *Ruppia* were characterized as many-seeded, a trait beneficial for bird-dispersal [8] whereas tetraploid *Ruppia* few-seeded with rhizome mediated clonal growth, thus potentially also sea current dispersed [8]. However, we considered actual currents that are not exactly similar to historical sea currents e.g. during the relatively short period of sea level rise and faster changing coastlines following the LGM. The high levels of dispersal and cpDNA similarity along Atlantic coast and Baltic lagoons can be the result of more than one dispersal vector and repeated dispersal events. On basis of fossil evidence from seeds in recent deposits it was mentioned that *Ruppia* followed transgressing shore lines along the North sea and British Isles and that *R. maritima* was present in northern parts of the Baltic Sea showing a larger postglacial range than today, most likely caused by salinity decrease since the Littorina period [68]. Also in *Zostera marina* a high microsatellite allelic diversity was reported along the North Sea and southwest Baltic Sea [13]. Birds might have dispersed *Ruppia* seeds at various distances but they did not shape a continent-wide genetic structure and certainly did not homogenize the cpDNA pattern in the peri-Mediterranean across the subsequent glacial and interglacial periods. The substructure of the *R. cirrhosa complex* and vicariance as known for *Posidonia* and other West-East divergences [4], [5], [8] suggests a dispersal mode through sea currents with sharper boundaries between subbasins, faults and coastlines as a result (except for Adriatic, shallower parts of Aegean and nearby the Siculo-Tunesian Strait).

### LDD misconceptions

Unfortunately, cpDNA does not allow to disentangle flow versus fowl-mediated dispersal along coastlines of the Atlantic/Baltic or the Adriatic Sea. A seagrass species (*Thalassia testudinum* Banks & Sol. ex K.D. Koenig) with high dispersal potential through its traits such as drifting propagules, also showed IBD with significant differentiation at an estimated distance of more than 350 km [69]. Similarly, an isolation-by-distance was estimated at ∼150 km among northern European *Zostera marina* populations [13]. This albeit can be regarded as a limited coastline stretch along an entire continent when considering multiple dispersal events over historical periods in metapopulation settings. Long distance dispersal over entire or parts of continents (thousands of km) for a similar haplotype such as observed in this study for *R. cirrhosa* (haplotype C1) and *R. maritima* (D1) are seldom observations. These need further confirmation from nuclear microsatellite estimations of connectivity. LDD through birds was assumed to explain a disjunct distribution of *Ruppia megacarpa* Mason across both hemispheres (Japan and Southern Australia), however based on partial and short rbcL sequences [36]. Analysis of their methodological aspects reveal that only a small part of the rbcL (542 bp) was used instead of the full sequence (>1091 bp). Large-scale disjunct distribution as a result of LDD thus remains questionable because when we limit the sequence length to the same as in [36], then our results would erroneously show that *R. maritima* populations appear similar from the Baltic Sea over the Mediterranean, Nile region to Southern Africa. This obviously is not supported by the full rbcL sequence we used, giving parsimonious information and divergences between Baltic, Mediterranean, tropical and southern African populations, thereby clearly rejecting the hypothesis of LDD across continents nearly spanning the globe in this example. To investigate more accurately the recent historical amount of gene flow and potential of LDD, it would be necessary to use SSRs from each region. However, prior to such study, accurate identification of the populations is needed because numerous misidentifications of especially *R. maritima* occur frequently and appear in literature, flora's as well as in Genbank [70]. In addition, LDD can hardly be proven when using loci that are only informative at species level (i.e. phylogenetic or barcoding) instead of the metapopulation level because such too slowly evolving marker loci rather may demonstrate the distribution area of a species than events of LDD.

More broadly, our study emphasizes the necessity of sequencing organel markers with various evolutionary rates to combine LDD hypotheses, shallow macroevolutionary phylogenies with historical microevolutionary processes. We conclude that both vicariance in the South (MSC to Pleistocene) and high seed flow in the North (LGM) can explain such vast species ranges.

Future conservation and restoration efforts should consider the possibility of cryptic taxa such as observed for *Zostera marina* and *Zostera pacifica* S.Watson [67]. In coastal areas of the Mediterranean basin, the pollution from land accounts for the majority of impacts [71]. Counter intuitive the most widespread seagrass *Ruppia* actually should be regarded as the most threatened of all euryhaline species and seagrasses because of direct habitat loss through coastal development of lagoons, associated saltmarshes, temporary pools and even artificial salinas with associated saltwater canals, this at a much larger rate than loss of strictly but open marine habitats. More damaging for *Ruppia* than for all related genera will be the hypereutrophication of lagoons and climate change where local conditions might create too short periods of ephemeral habitats behind the coastline and *Ruppia* populations getting locally extinct. Hotspots of diversity often occur at regional scale whereas at local scale more evidence is gained on the benefits from increased genetic diversity that might enhance various ecosystem services such as nutrient retention, biomass and support habitat for invertebrates [72] and wildlife [1]. Our findings highlight the need for interpreting global distributions of seagrass species with careful criticism in the context of their origin for setting up appropriate conservation strategies and for detecting diversity hotspots.

## Supporting Information

Figure S1 **Haplotype frequency distribution (group A, E).** *Ruppia drepanensis* (haplogroup A) and hybrid lineages of haplogroup E) of the *Ruppia cirrhosa* complex at site level in the peri-Mediterranean. The size of pie charts is relative to the sample size.
(TIF)

Figure S2 **Haplotype frequency distribution (group B, C).** *Ruppia cirrhosa* (haplogroup B and C) at site level across Europe and the peri-Mediterranean. The size of pie charts is relative to the sample size.
(TIF)

Figure S3 **Haplotype frequency distribution (group D).** *Ruppia maritima* (haplogroup D) at site level across Europe and the peri-Mediterranean. Hybrid populations with nuclear DNA of *R. maritima but* haplotype of *R. cirrhosa* are included. The size of pie charts is relative to the sample size.
(TIF)

Figure S4 **rbcL data: Maximum Likelihood Tree of seagrasses.** The *Ruppia cirrhosa* complex (Haplogroup A, B, C, E) and *Ruppia maritima* complex (Haplogroup D) from Europe and Africa diverged following the Messinian Salinity Crisis. *Acorus calamus* was used as outgroup. An overview of all taxa and Genbank numbers is provided in Table S3.
(TIF)

Table S1 **Collection localities.** A total of 2221 individuals of the *Ruppia cirrhosa* complex (Part A) and 622 of the *Ruppia maritima* complex (Part B) in Europe, Mediterranean and Africa (N = number of plants; cpDNA haplotypes and nuclear ITS as given in text and figures; ploidy level as inferred from duplicated microsatellite locus; BRVU refers to herbarium vouchers deposited at the Herbarium of the Vrije Universiteit Brussel; * refers to population collections made in 1983–1985 by the author and deposited at BRVU); § refers to collections used in earlier publications for only five [7], [8] instead of eleven cpDNA loci.
(DOCX)

Table S2 **New primers for chloroplast introns.** Designed from the *Acorus calamus* chloroplast genome. Six primers reveal variable regions between the haplotype lineages of *Ruppia maritima* (D), *R. drepanensis* (A) and the ancient *R. cirrhosa* hybrid complex (E). One duplication, a mononucleotide A-repeat, and especially the parsimonious information from transversions (tv) and transitions (ts) allowed to better separate the older lineages from *R. cirrhosa*.
(DOCX)

Table S3 **Overview of GenBank accession numbers.** A: GenBank accession numbers of seventeen Acmp intron sequences (newly developed primers from the *Acorus calamus* chloroplast genome as given in Table S2), corresponding to the *Ruppia* haplotype A–E variants as used in this study; B: GenBank accession numbers of *Ruppia* ccmp2, ccmp3, ccmp10, trnH-psbA, rbcL and ITS1-ITS2; : GenBank accession numbers of *rbcL* used in phylogenetic analysis of 18 seagrasses and related aquatics with *Acorus calamus* as an outgroup. JN113275–79 correspond to *Ruppia* haplotypes A–E variants as used in this study.
(DOCX)

Table S4 **Insertion-deletion events.** Overview of single duplication repeat motifs in cpDNA of *Ruppia maritima* (D). African D4 and D5 appear ancestral to European *R. maritima*. The Mediterranean *R. drepanensis* (A) and ancient *R. cirrhosa* hybrid complex (E) often are characterized by duplications. The Mediterranean *R. cirrhosa* (B) appears ancestral to the Atlantic and Baltic (C).
(DOCX)
