# Supplementary figures and images for: Seagrass Radiation after Messinian Salinity Crisis Reflected by Strong Genetic Structuring and Out-of-Africa Scenario (Ruppiaceae)
Source: PLoS One. 2014 Aug 6;9(8):e104264. doi: 10.1371/journal.pone.0104264 (PMC4123914; doi:10.1371/journal.pone.0104264)

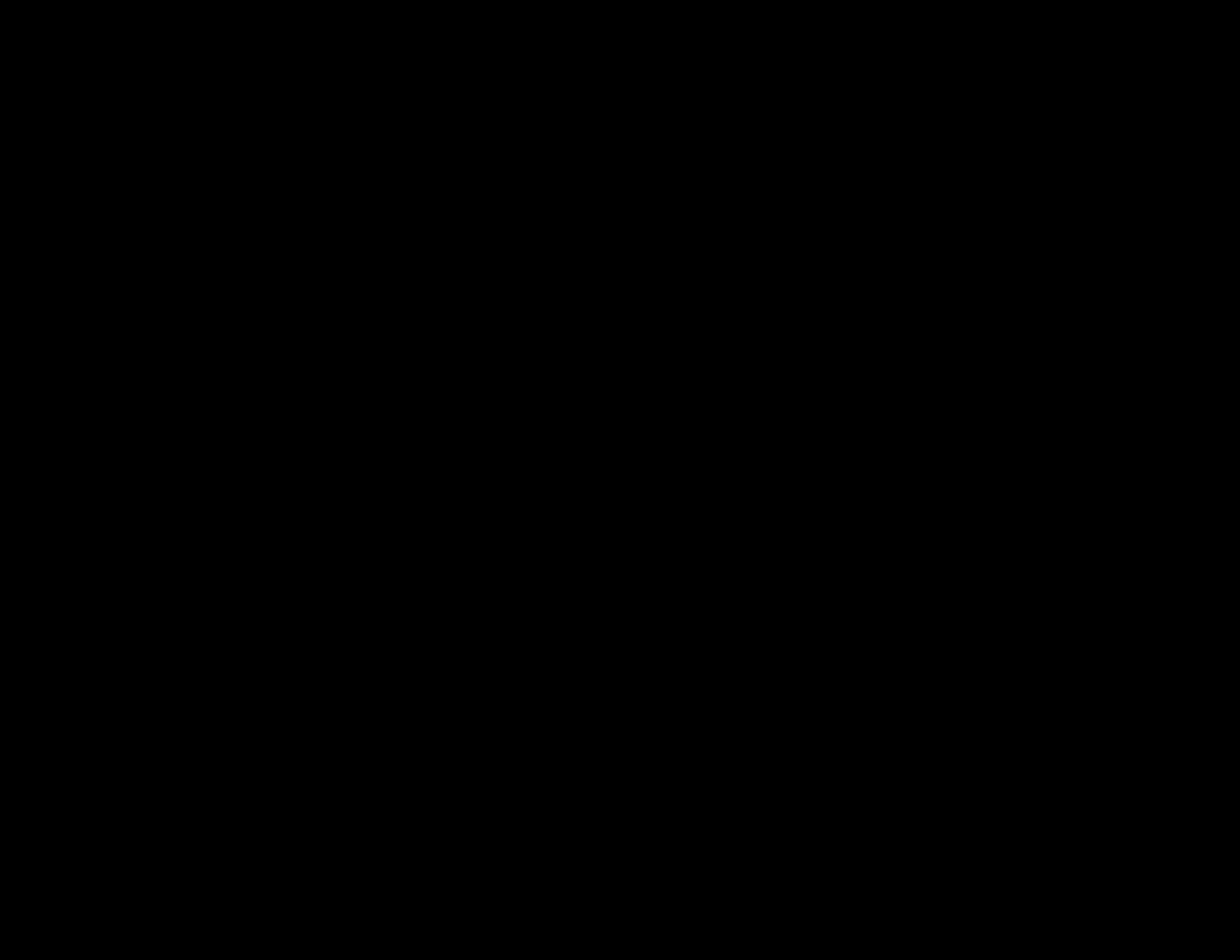

Supplement: Figure S1 — Haplotype frequency distribution (group A, E). Ruppia drepanensis (haplogroup A) and hybrid lineages of haplogroup E) of the Ruppia cirrhosa complex at site level in the peri-Mediterranean. The size of pie charts is relative to the sample size. (TIF) [file pone.0104264.s001.tif]

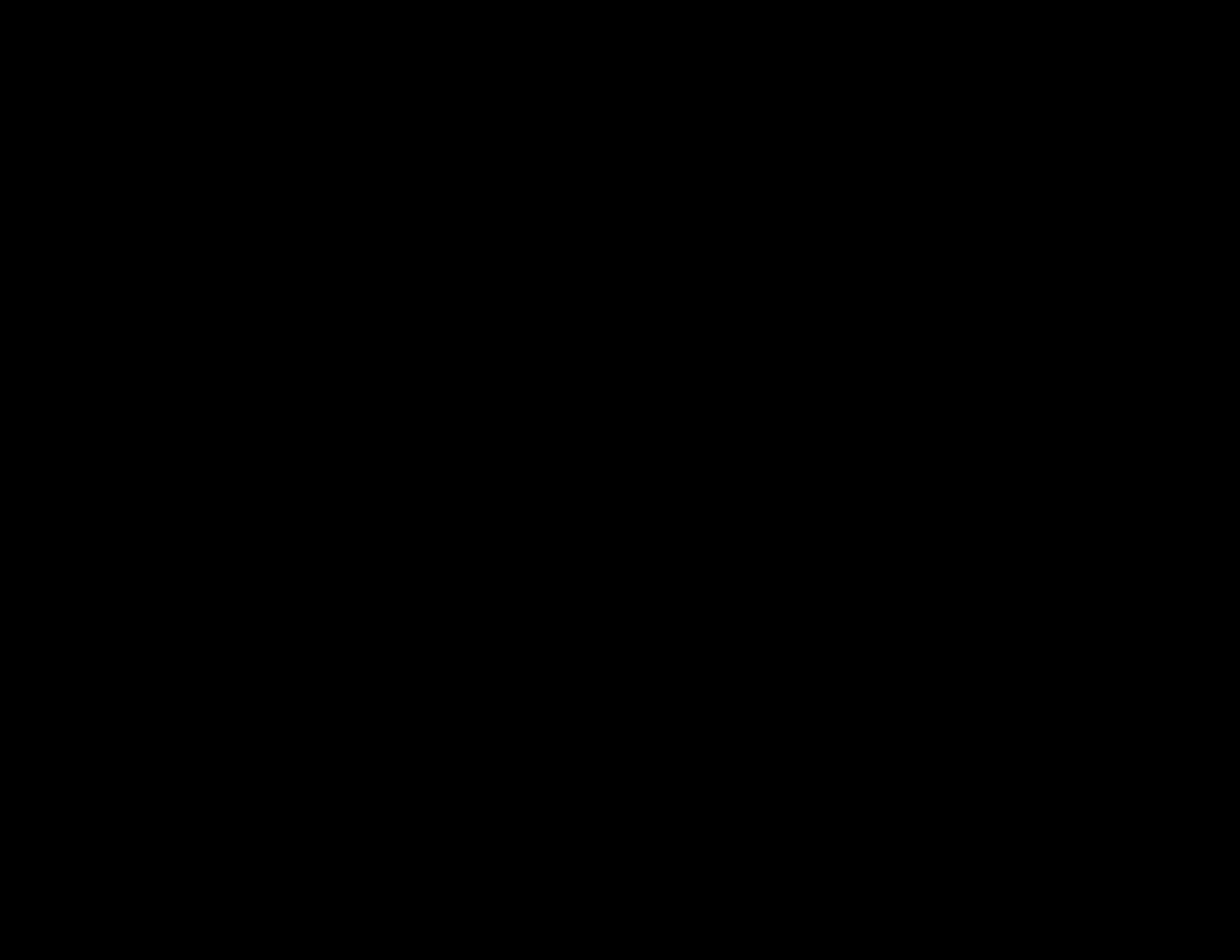

Supplement: Figure S2 — Haplotype frequency distribution (group B, C). Ruppia cirrhosa (haplogroup B and C) at site level across Europe and the peri-Mediterranean. The size of pie charts is relative to the sample size. (TIF) [file pone.0104264.s002.tif]

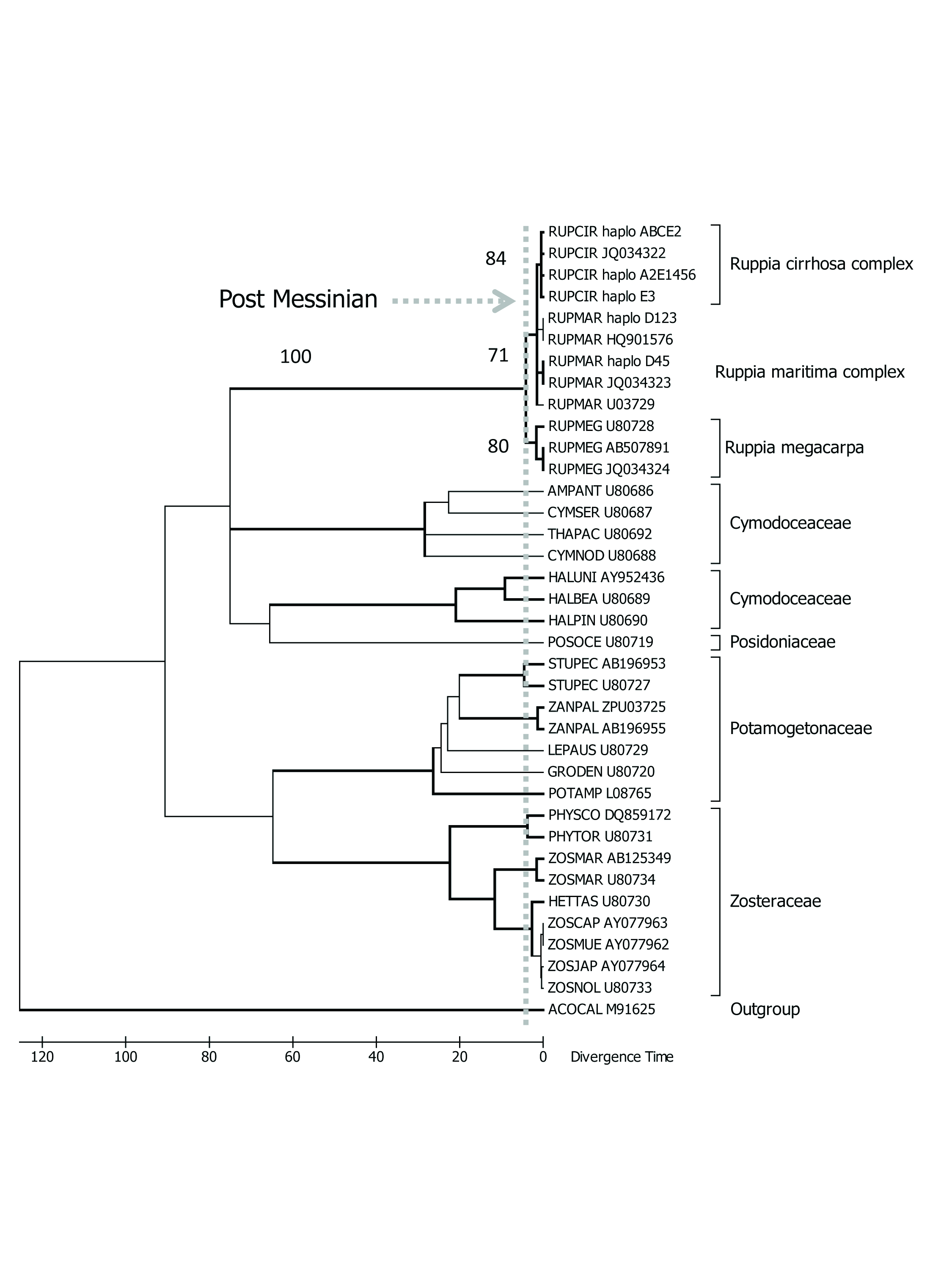

Supplement: Figure S4 — rbcL data: Maximum Likelihood Tree of seagrasses. The Ruppia cirrhosa complex (Haplogroup A, B, C, E) and Ruppia maritima complex (Haplogroup D) from Europe and Africa diverged following the Messinian Salinity Crisis. Acorus calamus was used as outgroup. An overview of all taxa and Genbank numbers is provided in Table S3. (TIF) [file pone.0104264.s004.tif]
