# Supplementary material for: Seagrass Radiation after Messinian Salinity Crisis Reflected by Strong Genetic Structuring and Out-of-Africa Scenario (Ruppiaceae)
Source: PLoS One. 2014 Aug 6;9(8):e104264. doi: 10.1371/journal.pone.0104264 (PMC4123914; doi:10.1371/journal.pone.0104264)
Supplement: Table S1 — Collection localities. A total of 2221 individuals of the Ruppia cirrhosa complex (Part A) and 622 of the Ruppia maritima complex (Part B) in Europe, Mediterranean and Africa (N = number of plants; cpDNA haplotypes and nuclear ITS as given in text and figures; ploidy level as inferred from duplicated microsatellite locus; BRVU refers to herbarium vouchers deposited at the Herbarium of the Vrije Universiteit Brussel; * refers to population collections made in 1983–1985 by the author and deposited at BRVU); § refers to collections used in earlier publications for only five [7], [8] instead of eleven cpDNA loci. (DOCX) [file pone.0104264.s005.docx]

**Table S1.** **Collection localities**. A total of 2221 individuals of the *Ruppia cirrhosa* complex (Part A) and 622 of the *Ruppia maritima* complex (Part B) in Europe, Mediterranean and Africa (N = number of plants; cpDNA haplotypes and nuclear ITS as given in text and figures; ploidy level as inferred from duplicated microsatellite locus; BRVU refers to herbarium vouchers deposited at the Herbarium of the Vrije Universiteit Brussel; * refers to population collections made in 1983-1985 by the author and deposited at BRVU); § refers to collections used in earlier publications for only five [7],[8] instead of eleven cpDNA loci.

1. ***Ruppia cirrhosa* haplotype complex**

| **Locality / coastal or inland lake** | **Country** | **Long (E)** | **Lat (N)** | **N** | **Haplotypes** | **ITS** | **Ploidy** | **Collection** |
| --- | --- | --- | --- | --- | --- | --- | --- | --- |
| NORTHERN BALTIC |  |  |  |  |  |  |  |  |
| Turku region, Kustavi | Finland | 21,269 | 60,573 | 30 | C1 | ITS-B | 4x | 2006-2010 |
| Uusima, Kolaviken | Finland | 23,017 | 59,819 | 10 | C1 |  |  | BRVU-OR22* |
| Aland, Mariehamn | Finland | 19,881 | 60,105 | 10 | C1 |  |  | BRVU-OR20* |
| OSTSEE / SOUTHERN BALTIC |  |  |  |  |  |  |  |  |
| Mecklenburg-Vorpommern, Hiddensee, Kloster, Enddorn | Germany | 13,138 | 54,596 | 5 | C3 | ITS-B | 4x | 2006-2010 |
| Mecklenburg-Vorpommern, Hiddensee, Kloster, Schwedenhagen | Germany | 13,125 | 54,584 | 5 | C1 | ITS-B | 4x | 2006-2010 |
| Mecklenburg-Vorpommern, Hiddensee, Vitte, Furt | Germany | 13,114 | 54,545 | 30 | C1, C2, C5 | ITS-B | 4x | 2006-2010 |
| Mecklenburg-Vorpommern, Wustrow, Rerik | Germany | 11,611 | 54,103 | 29 | B1, C1, C5 | ITS-B | 4x | 2006-2010 |
| Schleswig-Holstein, Aussere Schlei | Germany | 9,926 | 54,459 | 3 | C1 | ITS-B |  | BRVU-RH32 |
| Gotland, Mariager Fjord | Denmark | 10,216 | 56,693 | 10 | C1 |  |  | BRVU-OR18* |
| NORTH SEA |  |  |  |  |  |  |  |  |
| Texel, Ottersaat | Netherlands | 4,864 | 53,051 | 20 | C1 |  |  | BRVU-OR2* |
| Texel, Dijkmanshuizen | Netherlands | 4,872 | 53,060 | 10 | C1 |  |  | BRVU-OR3* |
| Texel, Oudeschild | Netherlands | 4,878 | 53,067 | 10 | C1 |  |  | BRVU-OR4* |
| Texel, Nieuweschild | Netherlands | 4,885 | 53,073 | 10 | C1 |  |  | BRVU-OR5* |
| Texel, Wagelot | Netherlands | 4,897 | 53,093 | 10 | C1 |  |  | BRVU-OR6* |
| Texel, Noorden | Netherlands | 4,897 | 53,102 | 10 | C1 |  |  | BRVU-OR8* |
| Noord-Holland, Petten | Netherlands | 4,666 | 52,770 | 20 | C1 |  |  | BRVU-OR10* |
| Zeeland, Sirjansland | Netherlands | 4,017 | 51,680 | 30 | C1 | ITS-B | 4x | 2006-2010 |
| Nord PDC, Platier d'Oye | France | 2,082 | 51,007 | 30 | C1 | ITS-B | 4x | 2006-2010 |
| Nord PDC, Le Fort Vert 1 | France | 1,942 | 50,986 | 30 | C1 | ITS-B | 4x | 2006-2010 |
| Nord PDC, Le Fort Vert 2 | France | 1,946 | 50,986 | 30 | C1 | ITS-B | 4x | 2006-2010 |
| ATLANTIC |  |  |  |  |  |  |  |  |
| Charente Maritime, Hiers Brouage | France | -1,092 | 45,877 | 3 | B2 |  |  | BRVU-RH4 |
| Aquitaine, Audenge, Graveyron1 | France | -1,034 | 44,685 | 30 | C1 cp-capture | ITS-A | 4x | 2006-2010 |
| Aquitaine, Audenge, Graveyron2 | France | -1,042 | 44,689 | 30 | C1 cp-capture | ITS-A | 4x | 2006-2010 |
| Aquitaine, Audenge, Certes1 | France | -1,017 | 44,677 | 30 | C1 | ITS-B | 4x | 2006-2010 |
| Aquitaine, Audenge, Certes2 | France | -1,019 | 44,672 | 30 | C1 | ITS-B | 4x | 2006-2010 |
| Aquitaine, Lège-Cap Ferret, Reservoir de Pirhaillan | France | -1,223 | 44,711 | 30 | C1 | ITS-B | 4x | 2006-2010 |
| Aquitaine, Le Verdon-sur-Mer, Marais du Conseiller1 | France | -1,075 | 45,540 | 30 | C1 | ITS-B | 4x | 2006-2010 |
| Aquitaine, Le Verdon-sur-Mer, Marais du Conseiller2 | France | -1,073 | 45,540 | 30 | C1 | ITS-B | 4x | 2006-2010 |
| Aquitaine, Le Verdon-sur-Mer, Marais du Conseiller3 | France | -1,072 | 54,541 | 30 | C1 | ITS-B | 4x | 2006-2010 |
| INLAND SPAIN |  |  |  |  |  |  |  |  |
| Laguna de Manjavacas | Spain | -2,861 | 39,412 | 26 | A1, A2, A3, A4, A5 | ITS-C | 4x | 2006-2010§ |
| NP Donana , Valverde | Spain | -6,272 | 37,070 | 30 | A1 | ITS-C | 4x | 2006-2010§ |
| NP Donana , Valverde | Spain | -6,288 | 37,071 | 30 | A1 | ITS-C | 4x | 2006-2010§ |
| NP Donana , Valverde | Spain | -6,304 | 37,072 | 30 | B1 cp-capture | ITS-A | 4x | 2006-2010§ |
| ALBORAN / ALGERIAN |  |  |  |  |  |  |  |  |
| Oran, El Macta | Algeria | -0,124 | 35,773 | 3 | B1 |  |  | BRVU-RH9 |
| Between Guardias Viljas and Almerimar | Spain | -2,816 | 36,712 | 30 | B1 | ITS-B | 4x | 2006-2010§ |
|  |  |  |  |  | E1 cp-capture | ITS-A | 2x | 2006-2010§ |
| NP Salinas, Roquetas de Mar | Spain | -2,653 | 36,705 | 28 | B1 | ITS-B | 4x | 2006-2010§ |
|  |  |  |  |  | E1 cp-capture | ITS-A | 2x | 2006-2010§ |
| NP Salinas, Roquetas de Mar | Spain | -2,644 | 36,716 | 30 | C1 | ITS-B | 4x | 2006-2010§ |
| Between Torre de Pinet and St Pola | Spain | -0,613 | 38,184 | 30 | C1 | ITS-B | 4x | 2006-2010§ |
| Between Torre de Pinet and St Pola | Spain | -0,614 | 38,184 | 30 | B1, C1, C2, C4 | ITS-B | 4x | 2006-2010§ |
| Between Torre de Pinet and St Pola | Spain | -0,615 | 38,185 | 30 | C1 | ITS-B | 4x | 2006-2010§ |
| BALEARIC |  |  |  |  |  |  |  |  |
| Valencia | Spain | -0,320 | 39,322 | 3 | B1 |  |  | BRVU-RH6 |
| Albufera NP, Marina lagoon,Valencia | Spain | -0,315 | 39,346 | 30 | B1 | ITS-B | 4x | 2006-2010§ |
| Marjal des Moros, Puçol | Spain | -0,258 | 39,616 | 30 | B1 cp-capture | ITS-A | 4x | 2006-2010§ |
| Podonue, Bassa de la Tancada, delta de l'Ebre | Spain | 0,854 | 40,686 | 25 | C1,C2 | ITS-B | 4x | 2006-2010§ |
| Estartit | Spain | 3,193 | 42,031 | 30 | C1 | ITS-B | 4x | 2006-2010§ |
| NP Aiguemolls near Rosas | Spain | 3,115 | 42,233 | 30 | C1 | ITS-B | 4x | 2006-2010§ |
| Estartit | Spain | 3,190 | 42,029 | 30 | C1 | ITS-B | 4x | 2006-2010§ |
| Camargue, La Pallisade, Trou de l'oie | France | 4,814 | 43,363 | 30 | B1, C1, C2, C3 | ITS-B | 4x | 2006-2010§ |
| Camarge, La Pallisade, Le capouillet | France | 4,810 | 43,362 | 29 | C1, C2, C3 | ITS-B | 4x | 2006-2010§ |
| Between Carnon and La Grande Motte | France | 3,995 | 43,552 | 23 | B1, C1, C2, C3 | ITS-B | 4x | 2006-2010§ |
| Bouches du Rhone, Etang dit l’Imperial | France | 4,440 | 43,489 | 10 | C2 |  |  | BRVU-OR12* |
| Bouches du Rhone, Vaccares | France | 4,456 | 43,474 | 10 | C1 |  |  | BRVU-OR13* |
| Bouches du Rhone Cacharel | France | 4,655 | 43,521 | 10 | C1 |  |  | BRVU-OR14* |
| Camargue, Salin de Giraud, Le Sambuc | France | 4,806 | 43,375 | 10 | E6 |  |  | BRVU-OR15* |
| Camargue, Salin de Giraud, La Palissade | France | 4,807 | 43,374 | 10 | C1 |  |  | BRVU-OR16* |
| Menorca, Es Grau, saltmarsh transect along path | Spain | 4,265 | 39,946 | 29 | B4, C1 | ITS-B | 4x | 2006-2010§ |
| Menorca, Es Grau, Albufera | Spain | 4,263 | 39,947 | 30 | C1 | ITS-B | 4x | 2006-2010§ |
| Menorca, Salinas Mongofre | Spain | 4,206 | 39,988 | 29 | B1 cp-capture, E4 cp-capture | ITS-A | 4x | 2006-2010§ |
| Sardegna, Oristano, Santa Giusta | Italy | 8,609 | 39,872 | 30 | B1, B3, C1 | ITS-B | 4x | 2006-2010§ |
| Sardegna, Stagno Istai | Italy | 8,461 | 39,970 | 27 | B1, B3, C1 | ITS-B |  | 2006-2010§ |
| TYRRHENIC |  |  |  |  |  |  |  |  |
| Corsica, Ghisonaccia | France | 9,479 | 42,040 | 10 | C1 |  |  | BRVU-ORC* |
| Borgo Grappa, NP Circeo | Italy | 12,925 | 41,384 | 30 | B1,B2 | ITS-B | 4x | 2006-2010§ |
| Foglianu, NP Circeo | Italy | 12,920 | 41,388 | 30 | B1 | ITS-B | 4x | 2006-2010§ |
| Orbetello lagoon | Italy | 11,243 | 42,427 | 30 | C1 | ITS-B | 4x | 2006-2010§ |
| Castiglione della Pescaia | Italy | 10,898 | 42,764 | 30 | E3 cp-capture | ITS-A | 2x | 2006-2010§ |
| Castiglione della Pescaia, Badiola | Italy | 10,940 | 42,782 | 30 | C1 | ITS-B | 4x | 2006-2010§ |
| Sardegna, Cagliari, W-lagoon, salinas | Italy | 9,024 | 39,181 | 30 | B1, C1 | ITS-B | 4x | 2006-2010§ |
| Sardegna, Chia, Monte Cogoni | Italy | 8,877 | 38,895 | 30 | B1, B2, C1 | ITS-B | 4x | 2006-2010§ |
| Sardegna, Su Giudeu | Italy | 8,868 | 38,889 | 30 | B1, B2, C1 | ITS-B | 4x | 2006-2010§ |
|  |  |  |  |  | E3 | ITS-A | 2x | 2006-2010§ |
| Sardegna, Porto Corallo lagoon | Italy | 9,618 | 39,435 | 30 | B2 | ITS-B | 4x | 2006-2010§ |
| Sicily, Palermo, Mondello | Italy | 13,324 | 38,211 | 3 | B1 |  |  | BRVU-RH7 |
| Sicily, Trapani saltmarsh location 1 | Italy | 12,485 | 37,860 | 10 | B2 | ITS-B | 4x | 2006-2010§ |
| Sicily, Trapani saltmarsh location 2 | Italy | 12,485 | 37,868 | 10 | B1 | ITS-B | 4x | 2006-2010§ |
| Sicily, Trapani saltmarsh location 3 | Italy | 12,486 | 37,869 | 10 | B1 | ITS-B | 4x | 2006-2010§ |
| Djerba, Sidi Yati | Tunesia | 10,975 | 33,732 | 3 | B1 |  |  | BRVU-RH12 |
| ADRIATIC |  |  |  |  |  |  |  | 2006-2010§ |
| Grado, NR Valle Cavanata | Italy | 13,476 | 45,715 | 30 | B1, B2 | ITS-B | 4x | 2006-2010§ |
| Valle di Comacchia | Italy | 12,245 | 44,580 | 26 | B1, B2, C1 | ITS-B | 4x | 2006-2010§ |
| Portoroz, Secovlje salina 1 | Slovenia | 13,608 | 45,492 | 30 | B1 | ITS-B | 4x | 2006-2010§ |
| Portoroz, Small salina | Slovenia | 13,609 | 45,528 | 30 | B1 | ITS-B | 4x | 2006-2010§ |
| Portoroz, Secovlje salina 3 | Slovenia | 13,608 | 45,491 | 16 | B1 | ITS-B | 4x | 2006-2010§ |
| IONIAN |  |  |  |  |  |  |  |  |
| Arta, Logarou | Greece | 20,924 | 39,013 | 30 | B1 | ITS-B | 4x | 2006-2010§ |
| Arta, Logarou | Greece | 20,929 | 39,017 | 30 | B1 | ITS-B | 4x | 2006-2010§ |
| Arta, Logarou, Koronissiu | Greece | 20,849 | 39,033 | 30 | B1 | ITS-B | 4x | 2006-2010§ |
| Arta, Logarou, Lake Tsoukalio | Greece | 20,874 | 39,061 | 30 | B1, E2 | ITS-B | 4x | 2006-2010§ |
| Messolonghi | Greece | 21,429 | 38,333 | 30 | B1 | ITS-B | 4x | 2006-2010§ |
| Messolonghi | Greece | 21,432 | 38,332 | 30 | B1 | ITS-B | 4x | 2006-2010§ |
| Ilia, Lake Kotychi | Greece | 21,386 | 38,160 | 30 | B1 | ITS-B | 4x | 2006-2010§ |
| Ilia, Lake Kotychi | Greece | 21,387 | 38,151 | 30 | B1 | ITS-B | 4x | 2006-2010§ |
| Achaia, Lake Prokopos | Greece | 21,288 | 38,005 | 30 | B1 | ITS-B | 4x | 2006-2010§ |
| Achaia, Lake Prokopos | Greece | 21,282 | 37,996 | 30 | B1 | ITS-B | 4x | 2006-2010§ |
| Aigio, Aliki lagoon | Greece | 22,108 | 38,265 | 1 | B2 | ITS-B |  | 2006-2010§ |
| NORTHERN AEGEAN |  |  |  |  |  |  |  |  |
| Evros, Monolimni B2_reproductive | Greece | 26,064 | 40,774 | 15 | B1 | ITS-B | 4x | 2006-2010§ |
| Evros, Monolimni B2_vegetative | Greece | 26,053 | 40,777 | 15 | B1 | ITS-B | 4x | 2006-2010§ |
| Evros, Drana Station DC | Greece | 26,021 | 40,807 | 6 | B1, B2 | ITS-B | 4x | 2006-2010§ |
| LEVANTINE / NILE / MIDDLE EAST |  |  |  |  |  |  |  |  |
| Oasis de Koufra | Libia | 23,276 | 24,19 | 3 | E1 |  |  | BRVU-RH14 |
| Tripolitana, Zaouia | Libia | 12,271 | 32,838 | 3 | A1 |  |  | BRVU-RH15 |
| Alexandria, Lake Idku | Egypt | 30,217 | 31,210 | 10 | B1 |  |  | BRVU-RH17* |
| Alexandria, Bourg-el-Arab | Egypt | 29,644 | 30,98 | 10 | E1 |  |  | BRVU-RH21B* |
| Alexandria | Egypt | 29,430 | 30,895 | 10 | E1 |  |  | BRVU-RH23* |
| Lake Burullus, S. of Baltim | Egypt | 31,069 | 31,528 | 10 | E1 |  |  | BRVU-RH19* |
| Near Famagusta | Cyprus | 33,973 | 35,325 | 3 | E1 | ITS-A |  | BRVU- RH24 |
| Merkaz Sapir | Israel | 35,190 | 30,616 | 10 | E1 | ITS-A |  | BRVU-RH25 |
| Fayoum, shore near Auberge | Egypt | 30,743 | 29,473 | 10 | B1 |  |  | BRVU-RH16* |
| Fayoum, 6 km W of Shaksouk | Egypt | 30,828 | 29,518 | 10 | E5 | ITS-A |  | BRVU-RH18* |
| Fayoum, near Shaksouk | Egypt | 30,817 | 29,533 | 10 | E6 | ITS-A |  | BRVU-RH20* |
| Fayoum, Kota Qarun | Egypt | 30,496 | 29,401 | 10 | E3 | ITS-A |  | BRVURH22* |

***B. Ruppia maritima* haplotype complex**

| **Locality (coastal or inland lake)** | **Country** | **Long (E)** | **Lat (N)** | **N** | **Haplotypes** | **ITS** | **Ploidy** | **Collection** |
| --- | --- | --- | --- | --- | --- | --- | --- | --- |
| NORTHERN BALTIC |  |  |  |  |  |  |  |  |
| Kustavi | Finland | 21,259 | 60,58 | 30 | D1 | ITS-A | 2x | 2006-2010 |
| Uusikaupunki | Finland | 21,307 | 60,822 | 30 | D1 | ITS-A | 2x | 2006-2010 |
| Uusikaupunki | Finland | 21,404 | 60,79 | 26 | D1, D2 | ITS-A | 2x | 2006-2010 |
| Vormsi | Estonia | 23,167 | 58,977 | 36 | D1, D3 | ITS-A | 2x | 2006-2010 |
| Aland, Mariehamn | Finland | 19,911 | 60,085 | 10 | D1 |  |  | BRVU-OR19* |
| Uusima, Tvärminne | Finland | 23,017 | 59,819 | 10 | D1 |  |  | BRVU-OR21* |
| Uusima, Kolaviken | Finland | 23,398 | 59,854 | 10 | D1 |  |  | BRVU-OR23* |
| OSTSEE / SOUTHERN BALTIC |  |  |  |  |  |  |  |  |
| Schleswig-Holstein, Aussere Schlei | Germany | 9,922 | 54,458 | 3 | D1 |  |  | BRVU-RH33 |
| Schleswig-Holstein, Aussere Schlei | Germany | 10,184 | 54,444 | 3 | D1 |  |  | BRVU-RH34 |
| NORTH SEA |  |  |  |  |  |  |  |  |
| Texel, Wagelot | Netherlands | 4,898 | 53,093 | 10 | D1 |  |  | BRVU-OR27* |
| Texel, Wagelot | Netherlands | 4,899 | 53,089 | 10 | D1 |  |  | BRVU-OR7* |
| Texel, Nieuweschild | Netherlands | 4,885 | 53,073 | 10 | D1 |  |  | BRVU-OR9* |
| Zeeland, Nieuwe Naters | Netherlands | 3,971 | 51,723 | 10 | D1 |  |  | BRVU-OR1* |
| Nieuwpoort | Belgium | 2,739 | 51,148 | 3 | D1 |  |  | BRVU-RH2 |
| Dieppe, Tréport | France | 1,383 | 50,061 | 3 | D1 |  |  | BRVU-RH3 |
| Bourbourg | France | 2,155 | 50,93 | 29 | D1 | ITS-A | 2x | 2006-2010 |
| ATLANTIC |  |  |  |  |  |  |  |  |
| Le Teich | France | -1,011 | 44,647 | 30 | D1 | ITS-A | 2x | 2006-2010 |
| Le Teich | France | -1,024 | 44,654 | 30 | D1 |  | 2x | 2006-2010 |
| Le Teich | France | -1,029 | 44,655 | 30 | D1 |  | 2x | 2006-2010 |
| St Jean de luz | France | -1,645 | 43,378 | 31 | D1 | ITS-A | 2x | 2006-2010 |
| St Jean de luz | France | -1,645 | 43,377 | 26 | D1 |  | 2x | 2006-2010 |
| INLAND CENTRAL EUROPE |  |  |  |  |  |  |  |  |
| Marsal | France | 6,606 | 48,79 | 30 | D1 | ITS-A | 2x | 2006-2010 |
| Artern | Germany | 11,284 | 51,371 | 30 | D1 | ITS-A | 2x | 2006-2010 |
| Artern | Germany | 11,283 | 51,369 | 30 | D1 |  | 2x | 2006-2010 |
| Artern | Germany | 11,284 | 51,369 | 30 | D1 |  | 2x | 2006-2010 |
| BALEARIC |  |  |  |  |  |  |  |  |
| Torreblanca | Spain | 0,215 | 40,189 | 15 | D1 | ITS-A | 2x | 2006-2010§ |
| Camargue, Salin de Giraud | France | 4,805 | 43,379 | 31 | D1 | ITS-A | 2x | 2006-2010§ |
| Camargue, Salin de Giraud | France | 4,809 | 43,372 | 10 | D1 |  |  | BRVU-OR17* |
| Son Bou | Spain | 4,069 | 39,902 | 30 | D1 | ITS-A | 2x | 2006-2010§ |
| LEVANTINE / NILE |  |  |  |  |  |  |  |  |
| Alexandria, Bourg-el-Arab | Egypt | 29,644 | 30,98 | 10 | D1 |  |  | BRVU-RH21* |
| TROPICAL AFRICA |  |  |  |  |  |  |  |  |
| Haut-Katanga, Mwashya | Congo | 27,337 | -10,704 | 3 | D4 |  |  | BRVU-RH28 |
| Haut-Katanga, Kalamato | Congo | 27.444 | -7.289 | 3 | D4 |  |  | BRVU-RH29 |
| Natal, St Lucia Bay | South Africa | 27,885 | -32,418 | 10 | D5 | ITS-A |  | BRVU-RH30* |
| Natal, St Lucia Bay pond | South Africa | 27,885 | -32,418 | 10 | D5 |  |  | BRVU-RH31* |
