# Supplementary material for: Seagrass Radiation after Messinian Salinity Crisis Reflected by Strong Genetic Structuring and Out-of-Africa Scenario (Ruppiaceae)
Source: PLoS One. 2014 Aug 6;9(8):e104264. doi: 10.1371/journal.pone.0104264 (PMC4123914; doi:10.1371/journal.pone.0104264)
Supplement: Table S2 — New primers for chloroplast introns. Designed from the Acorus calamus chloroplast genome. Six primers reveal variable regions between the haplotype lineages of Ruppia maritima (D), R. drepanensis (A) and the ancient R. cirrhosa hybrid complex (E). One duplication, a mononucleotide A-repeat, and especially the parsimonious information from transversions (tv) and transitions (ts) allowed to better separate the older lineages from R. cirrhosa. (DOCX) [file pone.0104264.s006.docx]

**Table S2. New primers for chloroplast introns.** Designed from the *Acorus calamus* chloroplast genome. Six primers reveal variable regions between the haplotype lineages of *Ruppia maritima* (D), *R. drepanensis* (A) and the ancient *R. cirrhosa* hybrid complex (E). One duplication, a mononucleotide A-repeat, and especially the parsimonious information from transversions (tv) and transitions (ts) allowed to better separate the older lineages from *R. cirrhosa*.

| primer name | primer sequence (5' - 3') | position in *Acorus calamus* chloroplast genome | | Gene | bp | Haplotype | Variant | Position |
| --- | --- | --- | --- | --- | --- | --- | --- | --- |
| acmp 1F | TAATTTCATCGGCTCGAAGG | 11760 | 11779 | atpA | 179 | D4 | C (tv) | 44 |
| acmp 1R | CATTTCCGTACCATCAGTGC | 11898 | 11917 | atpF | 175-179 | D | (A)7-10 | 94-97 |
|  |  |  |  |  |  |  |  |  |
| acmp 2F | CCTGTTCTTTCCATGACTCCTC | 35720 | 35741 | psbC | 169 | D1,2,3 | T (tv) | 30 |
| acmp 2R | GGGTTCGAATCCCTCTCTCT | 35972 | 35991 | tRNA-  Ser (UGA) |  |  |  |  |
|  |  |  |  |  |  |  |  |  |
| acmp 4F | CGCACCCAAAACATACCATT | 79665 | 79684 | infA | 228 | A2 | T (ts) | 122 |
| acmp 4R | GCTCGACTACAGGGAATTGG | 79895 | 79914 | rpS8 |  | D | A (ts) | 171 |
|  |  |  |  |  |  |  |  |  |
| acmp 5F | CCAAAGAGGAATCCTTCCAG | 63808 | 63827 | psbJ | 222 | D | A (ts) | 100 |
| acmp 5R | TGGGGGTTATTACTCATTTTTG | 64014 | 64035 | psbL |  | A,D,E | C (ts) | 104 |
|  |  |  |  |  |  |  |  |  |
| acmp 6F | CCGAAACATAAACAAAGACA | 79282 | 79301 | rpL36 | 209 | E5 | (CCTAAAA)2 | 33-49 |
| acmp 6R | CCAATACGTCCATTCCTACG | 79105 | 79124 | rpS11 | 202 | D | C (ts) | 59 |
|  |  |  |  |  |  |  |  |  |
| acmp 7F | TTTCCCTAGCTCCTGAGGTA | 80463 | 80444 | rpL14 | 194 | D | A (ts) | 86 |
| acmp 7R | CGGCAATAGTGCCTTACCC | 80263 | 80244 | rpS8 |  |  |  |  |

Genbank accession numbers deposited for variants in *Ruppia* of Acmp1 (KJ010058, KJ010059, KJ010060, KJ010061), Acmp2 (KJ010062, KJ010063), Acmp4 (KJ010064, KJ010065, KJ010066), Acmp5 (KJ010067, KJ010068, KJ010069), Acmp6 (KJ010070, KJ010071, KJ010072), Acmp7 (KJ010073, KJ010074).
