# Supplementary material for: Seagrass Radiation after Messinian Salinity Crisis Reflected by Strong Genetic Structuring and Out-of-Africa Scenario (Ruppiaceae)
Source: PLoS One. 2014 Aug 6;9(8):e104264. doi: 10.1371/journal.pone.0104264 (PMC4123914; doi:10.1371/journal.pone.0104264)
Supplement: Table S3 — Overview of GenBank accession numbers. A: GenBank accession numbers of seventeen Acmp intron sequences (newly developed primers from the Acorus calamus chloroplast genome as given in Table S2), corresponding to the Ruppia haplotype A–E variants as used in this study; B: GenBank accession numbers of Ruppia ccmp2, ccmp3, ccmp10, trnH-psbA, rbcL and ITS1-ITS2; : GenBank accession numbers of rbcL used in phylogenetic analysis of 18 seagrasses and related aquatics with Acorus calamus as an outgroup. JN113275–79 correspond to Ruppia haplotypes A–E variants as used in this study. (DOCX) [file pone.0104264.s007.docx]

**Table S3. Overview of GenBank accession numbers.** A: GenBank accession numbers of seventeen Acmp intron sequences (newly developed primers from the *Acorus calamus* chloroplast genome as given in Table S2), corresponding to the *Ruppia* haplotype A-E variants as used in this study; B: GenBank accession numbers of *Ruppia* ccmp2, ccmp3, ccmp10, trnH-psbA, rbcL and ITS1-ITS2; : GenBank accession numbers of *rbcL* used in phylogenetic analysis of 18 seagrasses and related aquatics with *Acorus calamus* as an outgroup. JN113275-79 correspond to *Ruppia* haplotypes A-E variants as used in this study.

A (From this study)

| GenBank Accession | Species and haplotype lineage | Haplotype | Locus |
| --- | --- | --- | --- |
| KJ010060 | *Ruppia maritima* (D) | D | Acmp1_178 |
| KJ010059 | *Ruppia maritima* (D3) | D3 | Acmp1_177 |
| KJ010061 | *Ruppia maritima* (D4 from Africa, Congo) | D4 | Acmp1_179 |
| KJ010058 | *Ruppia cirrhosa* (B, C, E) and *Ruppia drepanensis* (A) | A, B, C, E | Acmp1_175 |
| KJ010062 | *Ruppia drepanensis* (A), *Ruppia cirrhosa* (B, C), ancient hybrid *Ruppia cirrhosa* complex (E) and African *Ruppia maritima* (D4, D5) | A, B, C, D4, D5, E | Acmp2_169a |
| KJ010063 | *Ruppia maritima* ((D1, D2, D3 from Europe) | D1, D2, D3 | Acmp2_169b |
| KJ010064 | *Ruppia drepanensis* (A), *Ruppia cirrhosa* (B, C) and ancient hybrid *Ruppia cirrhosa* complex (E) | A, B, C, E | Acmp4_228a |
| KJ010065 | *Ruppia maritima* (D) | D | Acmp4_228b |
| J010066 | *Ruppia drepanensis* (A2) | A2 | Acmp4_228c |
| KJ010067 | *Ruppia cirrhosa (B, C and E2)* | B, C, E2 | Acmp5_222a |
| KJ010068 | *Ruppia drepanensis* (A)and ancient hybrid *Ruppia cirrhosa* complex (E) | A, E | Acmp5_222b |
| KJ010069 | *Ruppia maritima* (D) | D | Acmp5_222c |
| KJ010072 | *Ruppia maritima* (D) | D | Acmp6_202b |
| KJ010071 | *Ruppia drepanensis* (A), *Ruppia cirrhosa* (B, C)ancient hybrid *Ruppia cirrhosa* complex (E) | A, B, E | Acmp6_202a |
| KJ010070 | ancient hybrid *Ruppia cirrhosa* complex (E5) | E5 | Acmp6_209 |
| KJ010073 | *Ruppia drepanensis* (A), *Ruppia cirrhosa* (B, C) and ancient hybrid *Ruppia cirrhosa* complex (E) | A, B, C, E | Acmp7_194a |
| KJ010074 | *Ruppia maritima* (D) | D | Acmp7_194b |

B (From our previous studies [7],[8])

| GenBank Accession | Species | Haplotype | Locus |
| --- | --- | --- | --- |
| JN113249 | *Ruppia drepanensis* | A | ccmp2 |
| JN113250 | *Ruppia cirrhosa* | B | ccmp2 |
| JN113251 | *Ruppia cirrhosa* | B | ccmp2 |
| JN113252 | *Ruppia cirrhosa* | C | ccmp2 |
| JN113253 | *Ruppia cirrhosa* | C | ccmp2 |
| JN113254 | *Ruppia cirrhosa* | E | ccmp2 |
| JN113255 | *Ruppia maritima* | D | ccmp2 |
| JN113256 | *Ruppia cirrhosa* | C | ccmp2 |
| JN113257 | *Ruppia maritima* | D | ccmp3 |
| JN113258 | *Ruppia cirrhosa* | B | ccmp3 |
| JN113259 | *Ruppia cirrhosa* | E | ccmp3 |
| JN113260 | *Ruppia cirrhosa* | B | ccmp10 |
| JN113261 | *Ruppia maritima* | D | ccmp10 |
| JN113262 | *Ruppia cirrhosa* | E | ccmp10 |
| JN113263 | *Ruppia cirrhosa* | C | ccmp10 |
| JN113264 | *Ruppia drepanensis* | A | ccmp10 |
| JN113265 | *Ruppia drepanensis* | A | ccmp10 |
| JN113266 | *Ruppia drepanensis* | A | trnH-psbA |
| JN113267 | *Ruppia cirrhosa* | B | trnH-psbA |
| JN113268 | *Ruppia cirrhosa* | B | trnH-psbA |
| JN113269 | *Ruppia maritima* | D | trnH-psbA |
| JN113270 | *Ruppia cirrhosa* | E | trnH-psbA |
| JN113271 | *Ruppia cirrhosa* | E | trnH-psbA |
| JN113272 | *Ruppia maritima* | D | trnH-psbA |
| JN113273 | *Ruppia cirrhosa* | E | trnH-psbA |
| JN113274 | *Ruppia maritima* | D | trnH-psbA |
| JN113275 | *Ruppia cirrhosa* | B | rbcL |
| JN113276 | *Ruppia cirrhosa* | E | rbcL |
| JN113277 | *Ruppia cirrhosa* | E | rbcL |
| JN113278 | *Ruppia maritima* | D | rbcL |
| JN113279 | *Ruppia maritima* | D | rbcL |
| JN113280 | *Ruppia cirrhosa* | B | ITS1 |
| JN113281 | *Ruppia drepanensis* | A | ITS1 |
| JN113282 | *Ruppia maritima* | D | ITS1 |
| JN113283 | *Ruppia cirrhosa* | B | ITS2 |
| JN113284 | *Ruppia drepanensis* | A | ITS2 |
| JN113285 | *Ruppia maritima* | D | ITS2 |

C (from GenBank)

| GenBank Accession | Species | Family |
| --- | --- | --- |
| M91625 | *Acorus calamus* | Acoraceae |
| U80686 | *Amphibolis antarctica* | Cymodoceaceae |
| U80688 | *Cymodocea nodosa* | Cymodoceaceae |
| U80687 | *Cymodocea serrulata* | Cymodoceaceae |
| U80692 | *Thalassodendron pachyrhizum* | Cymodoceaceae |
| U80689 | *Halodule beaudettia* | Cymodoceaceae |
| U80690 | *Halodule pinifolia* | Cymodoceaceae |
| AY952436 | *Halodule uninervis* | Cymodoceaceae |
| U80719 | *Posidonia oceanica* | Posidoniaceae |
| AB196954 | *Groenlandia densa* | Potamogetonaceae |
| U80729 | *Lepilaena australis* | Potamogetonaceae |
| L08765 | *Potamogeton amplifolius* | Potamogetonaceae |
| U80727 | *Stuckenia pectinata (Potamogeton pectinatus)* | Potamogetonaceae |
| AB196953 | *Stuckenia pectinata (Potamogeton pectinatus)* | Potamogetonaceae |
| U03725 | *Zannichellia palustris* | Potamogetonaceae |
| AB196955 | *Zannichellia palustris* | Potamogetonaceae |
| JN113275 | *Ruppia drepanensis* and *Ruppia cirrhosa* (A, B, C, E2) | Ruppiaceae |
| JN113276 | *Ruppia drepanensis* and ancient hybrid *Ruppia cirrhosa* complex (A2, E1, E4, E5, E6) | Ruppiaceae |
| JN113277 | Ancient hybrid *Ruppia cirrhosa* complex (E3) | Ruppiaceae |
| JQ34322 | Ancient hybrid *Ruppia cirrhosa* complex | Ruppiaceae |
| U03729 | *Ruppia cf. maritima* | Ruppiaceae |
| JN113278 | *Ruppia maritima* (D1, D2, D3) | Ruppiaceae |
| JN113279 | *Ruppia maritima* (D4, D5) | Ruppiaceae |
| JQ034323 | *Ruppia maritima* | Ruppiaceae |
| HQ901576 | *Ruppia maritima* | Ruppiaceae |
| U80728 | *Ruppia megacarpa* | Ruppiaceae |
| AB507891 | *Ruppia megacarpa* | Ruppiaceae |
| JQ034324 | *Ruppia megacarpa* | Ruppiaceae |
| U80730 | *Heterozostera tasmanica* | Zosteraceae |
| DQ859172 | *Phyllospadix scouleri* | Zosteraceae |
| U80731 | *Phyllospadix torreyi* | Zosteraceae |
| AY077963 | *Zostera capricorni* | Zosteraceae |
| AY077964 | *Zostera japonica* | Zosteraceae |
| AB125349 | *Zostera marina* | Zosteraceae |
| U80734 | *Zostera marina* | Zosteraceae |
| AY077962 | *Zostera muelleri* | Zosteraceae |
| U80733 | *Zostera noltii* | Zosteraceae |
