# Supplementary material for: Seagrass Radiation after Messinian Salinity Crisis Reflected by Strong Genetic Structuring and Out-of-Africa Scenario (Ruppiaceae)
Source: PLoS One. 2014 Aug 6;9(8):e104264. doi: 10.1371/journal.pone.0104264 (PMC4123914; doi:10.1371/journal.pone.0104264)
Supplement: Table S4 — Insertion-deletion events. Overview of single duplication repeat motifs in cpDNA of Ruppia maritima (D). African D4 and D5 appear ancestral to European R. maritima. The Mediterranean R. drepanensis (A) and ancient R. cirrhosa hybrid complex (E) often are characterized by duplications. The Mediterranean R. cirrhosa (B) appears ancestral to the Atlantic and Baltic (C). (DOCX) [file pone.0104264.s008.docx]

**Table S4**. **Insertion-deletion events.** Overview of single duplication repeat motifs in cpDNA of *Ruppia maritima* (D). African D4 and D5 appear ancestral to European *R. maritima*. The Mediterranean *R. drepanensis* (A) and ancient *R. cirrhosa* hybrid complex (E) often are characterized by duplications. The Mediterranean *R. cirrhosa* (B) appears ancestral to the Atlantic and Baltic (C).

| Species (complex) | Region | Haplotype  with repeat | cpDNA | Position | Repeat motif |
| --- | --- | --- | --- | --- | --- |
| *R. maritima* | Europe and Africa | D | TrnH-psbA | 377-386 | (TA)5 |
| *R. maritima* | Europe and Africa | D | Ccmp2 | 137-142 | TAATGT |
| *R. maritima* | Europe | D1, 2, 3 | TrnH-psbA | 156-163 | ATTTTTTT |
| *R. maritima* | Baltic Sea (Finland) | D2 | TrnH-psbA | 107-127 | TAATATGAGTTTCTATTTAT |
| All species except *R. maritima* | Europe | A, B, C, E | TrnH-psbA | 227-231 | TATTA |
| *R. drepanensis* | Mediterranean (Spain) | A4 | Ccmp10 | 90-95 | CATGCG |
| *R. drepanensis* and ancient *R. cirrhosa* hybrid complex | Mediterranean | A5, E1, E2, E3, E4, E5 | Ccmp10 | 58-74 | AAAAAAC |
| Ancient *R. cirrhosa* hybrid complex | Mediterranean (Egypt) | E5 | Acp6 | 33-49 | CCTAAAA |
| Ancient *R. cirrhosa* hybrid complex | Europe | All except E3 | TrnH-psbA | 170-172 | ATT |
| *R. cirrhosa* | Baltic Sea (Germany) | C5 | Ccmp2 | 70-75 | TTTTTA |
| *R. cirrhosa* | Europe | C1, C2, C3, C4, C5 | Ccmp2 | 90-97 | TTTTATAC |
| *R. cirrhosa* | Mediterranean (Spain) | C4 | Ccmp10 | 147-154 | ATTTTAAA |
